# A multiplexed Cas13-based assay with point-of-care attributes for simultaneous COVID-19 diagnosis and variant surveillance

**DOI:** 10.1089/crispr.2022.0048

**Published:** 2022-11-11

**Authors:** Maturada Patchsung, Aimorn Homchan, Kanokpol Aphicho, Surased Suraritdechachai, Thanyapat Wanitchanon, Archiraya Pattama, Khomkrit Sappakhaw, Piyachat Meesawat, Thanakrit Wongsatit, Artittaya Athipanyasilp, Krittapas Jantarug, Niracha Athipanyasilp, Juthamas Buahom, Supapat Visanpattanasin, Nootaree Niljianskul, Pimchai Chaiyen, Ruchanok Tinikul, Nuanjun Wichukchinda, Surakameth Mahasirimongkol, Rujipas Sirijatuphat, Nasikarn Angkasekwinai, Michael A. Crone, Paul S. Freemont, Julia Joung, Alim Ladha, Omar Abudayyeh, Jonathan Gootenberg, Feng Zhang, Claire Chewapreecha, Sittinan Chanarat, Navin Horthongkham, Danaya Pakotiprapha, Chayasith Uttamapinant

**Affiliations:** 1 School of Biomolecular Science and Engineering, Vidyasirimedhi Institute of Science and Technology (VISTEC), Rayong, Thailand; 2 Department of Biochemistry and Center for Excellence in Protein and Enzyme Technology, Faculty of Science, Mahidol University, Bangkok, Thailand; 3 Department of Microbiology, Faculty of Medicine Siriraj Hospital, Mahidol University, Bangkok, Thailand; 4 PTT Public Company Limited, Bangkok, Thailand; 5 Division of Genomic Medicine and Innovation Support, Department of Medical Sciences, Ministry of Public Health, Nonthaburi, Thailand; 6 Department of Medicine, Faculty of Medicine Siriraj Hospital, Mahidol University, Bangkok, Thailand; 7 London Biofoundry, Imperial College Translation and Innovation Hub, White City Campus, 84 Wood Lane, London, UK; 8 Section of Structural and Synthetic Biology, Department of Infectious Disease, Imperial College London, London, UK; 9 UK Dementia Research Institute Centre for Care Research and Technology, Imperial College London, London, UK; 10 Howard Hughes Medical Institute, Cambridge, MA, USA; 11 Broad Institute of MIT and Harvard, Cambridge, MA, USA; 12 McGovern Institute for Brain Research at MIT, Cambridge, MA, USA; 13 Department of Biological Engineering, MIT, Cambridge, MA, USA; 14 Department of Brain and Cognitive Sciences, MIT, Cambridge, MA, USA; 15 Mahidol-Oxford Tropical Medicine Research Unit, Faculty of Tropical Medicine, Mahidol University, Bangkok, Thailand; 16 Wellcome Sanger Institute, Hinxton, UK

## Abstract

Point-of-care (POC) nucleic acid detection technologies are poised to aid gold-standard technologies in controlling the COVID-19 pandemic, yet shortcomings in the capability to perform critically needed complex detection—such as multiplexed detection for viral variant surveillance—may limit their widespread adoption. Herein, we developed a robust multiplexed CRISPR-based detection using LwaCas13a and PsmCas13b to simultaneously diagnose SARS-CoV-2 infection and pinpoint the causative SARS-CoV-2 variant of concern (VOC)—including globally dominant VOCs Delta (B.1.617.2) and Omicron (B.1.1.529)—all while maintaining high levels of accuracy upon the detection of multiple SARS-CoV-2 gene targets. The platform has several attributes suitable for POC use: premixed, freeze-dried reagents for easy use and storage; convenient direct-to-eye or smartphone-based readouts; and a one-pot variant of the multiplexed detection. To reduce reliance on proprietary reagents and enable sustainable use of such a technology in low- and middle-income countries, we locally produced and formulated our own recombinase polymerase amplification reaction and demonstrated its equivalent efficiency to commercial counterparts. Our tool—CRISPR-based detection for simultaneous COVID-19 diagnosis and variant surveillance which can be locally manufactured—may enable sustainable use of CRISPR diagnostics technologies for COVID-19 and other diseases in POC settings.

## Introduction

The recurrent emergence of new severe acute respiratory syndrome coronavirus 2 (SARS-CoV-2) variants emphasizes the need of broad genomic and variant surveillance to monitor SARS-CoV-2 characteristics and evolution. Genomic and variant surveillance is critical to the understanding of the public health risk posed by new variants, as well as to the re-design and continual improvements of vaccines, therapeutics, and diagnostic technologies. Low- and middle-income countries (LMICs) are hotspots where several SARS-CoV-2 variants of concern (VOCs) are postulated to emerge from^1^, yet LMICs are at a distinct disadvantage in ramping up variant surveillance capacity, due to resources being more limited, mismanaged, or both. The majority (77%) of LMICs sequenced a minuscule fraction (<0.5%) of their COVID-19 cases; 20 LMICs do not have any sequencing activity reported in public databases^1,2^. Together, this calls for a robust and affordable genomic surveillance for LMICs.

Beyond genomic/variant surveillance activity, LMICs have limited access to the newest crisis management technologies; lack the capacity and infrastructure to manufacture and distribute crisis-critical products; and rely heavily on imports of these products which are subjected to constraints in the supply chain. Efforts to strengthen genomic/variant surveillance activity of LMICs in a sustainable manner—through the development of simple-to-use surveillance tools that can be locally manufactured–should be a global priority. Ideally, diagnostics and genomic/variant surveillance should be combined in the same technological platform to maximize disease detection and variant surveillance capacity while optimizing resource use.

Genetic surveillance of SARS-CoV-2 can either target the whole viral genome or specific genetic variations such as single nucleotide polymorphisms and indels through the use of next-generation sequencing or quantitative real-time PCR with reverse transcription (RT-qPCR).^3^ However, both sequencing- and PCR-based investigations could be slow in returning results, thereby delaying an effort to contain the outbreak especially for VOCs with greater transmissibility such as Delta (B.1.617.2; R0 of 5-7)^4,5^ and Omicron (B.1.1.529; R0 estimated to be as high as 10).^6^ Most importantly, current surveillance technologies require complicated, expensive instruments and expertise in handling and data analysis, resulting in the limited use of these technologies in LMICs^2^ where disease detection and variant surveillance are critically needed.

The collateral activity of clustered regularly interspaced short palindromic repeats (CRISPR)-associated 12/13 (Cas12/13) nucleases^7–10^ has recently been used as a basis for nucleic acid detection, including that of SARS-CoV-2.^11–14^ CRISPR-based detection has many attributes suitable for use in LMICs especially in point-of-care settings: the assay does not need complicated equipment and provides fast-to-return results, while maintaining high levels of diagnostic accuracy and complex testing capabilities characteristic of laboratory-based diagnostic technologies. CRISPR-based detection is particularly amenable to multiplexing, due to orthogonal cleavage preferences of bystander nucleic acid probes by different Cas enzymes—particularly the Cas13 family—each of which can be programmed for sequence-specific detection by its corresponding crRNA.^8^ Due to its high sequence specificity (when carefully designed and/or optimized), CRISPR-based detection has been explored as a tool to map key SARS-CoV-2 mutations^15^. A few reported technologies use equipment-heavy PCR^16^ and Fluidigm microfluidics^17^ to amplify genetic materials and increase the assay throughput, respectively. While such technologies drastically enhance the ability to profile multiple mutations of SARS-CoV-2 and other viruses, the reliance on extensive equipment may exclude them from utility in resource-poor settings. Equipment-light CRISPR-based detection is considered highly practical for several reasons: these platforms often combine an isothermal amplification step—compatible with a simple heating apparatus like a water bath and obviating the need of a PCR thermocycler—with a CRISPR-Cas-based detection step for maximal sensitivity and specificity. While discrimination of SARS-CoV-2 VOCs can be achieved with POC CRISPR-based detection, current POC CRISPR-based assays have diminished sensitivity (a trade-off upon using a simplified protocol^18^ and lack multiplexed detection capability.^18,19^)

**Figure 1:**
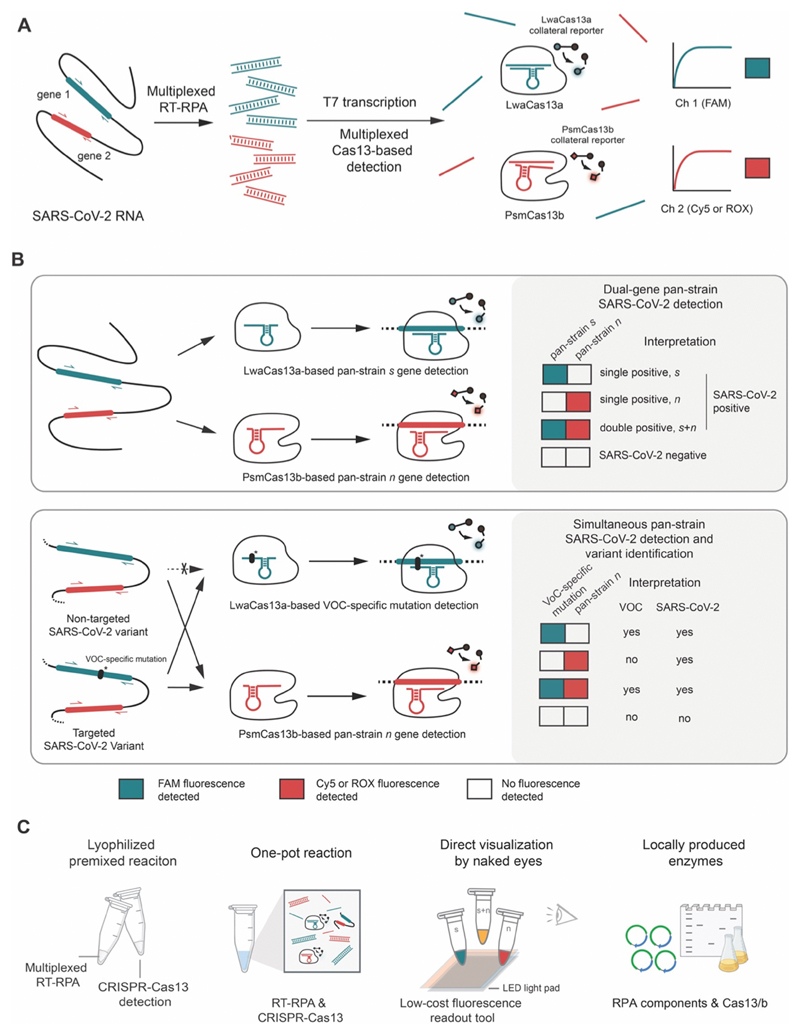
A multiplexed Cas13-based assay for simultaneous SARS-CoV-2 detection and variant identification. (**A**) Regions of interest in SARS-CoV-2 RNA, such as the *s* and *n* genes, are isothermally amplified by multiplexed RT-RPA. T7 transcription then converts and amplifies DNA amplicons to RNAs, which are recognized by cognate Cas13a-crRNA and Cas13b-crRNA complexes with orthogonal collateral cleavage preferences. Cleavage of orthogonal RNA reporters by target-activated Cas enzymes elicits multicolored fluorescence, which can be monitored with standard fluorescence detection instruments. (**B**) Top, dual-gene detection via CRISPR-Cas13a/b for COVID-19 diagnosis. Bottom, dual-gene detection for simultaneous COVID-19 diagnosis and SARS-CoV-2 variant identification. LwaCas13a is used to detect a universal, pan-strain region (top) or a variant-specific region (bottom) of the SARS-CoV-2 *s* gene. PsmCas13b is used to detect a pan-strain region of the *n* gene. The amplified regions of the S and N genes are shown in red and green respectively. (C) Developing simple-to-use SARS-CoV-2 variant surveillance tools which can be locally manufactured. Our multiplexed CRISPR-based detection reactions can be premixed and lyophilized for convenient use and storage; multiplexed RPA and Cas13-based reactions can be combined in one tube; result readouts can be directly observed by eye with low-cost light sources; and RPA and Cas13a/b can be locally produced and formulated.

Here we develop multiplexed CRISPR-based detection with point-of-care characteristics to clinically diagnose SARS-CoV-2 infection and inform the causative VOCs in the same reaction, while maintaining high sensitivity of detection (Figure 1). To do so, we extensively optimized components and reaction conditions of the reverse-transcription recombinase polymerase amplification (RT-RPA) to enable multiplexed amplification of two SARS-CoV-2 genes, *n* and *s* (Figure 1A, B). We explored several Cas13 enzymes including an engineered variant for multiplexed, orthogonal CRISPR-Cas13-based detection, and further screened different parameters for improved detection sensitivity. We lyophilized premixed amplification and CRISPR reactions and stored them in freeze-dried forms, which can be reconstituted simply through addition of buffered nucleic acid analytes. The freeze-dried reagent format enables convenient field deployment as well as longer reagent shelf-life. We further developed a highly sensitive one-pot reaction which combines multiplexed RT-RPA and Cas13-based detection in the same tube and demonstrated a simple visualization setup for multiplexed gene detection. Finally, our system was successfully validated in suspected SARS-CoV-2 samples from the real clinical setting; an indicative of both feasibility and robustness of our multiplexed Cas13-based assay.

After assessing cross-reactivity and the limit of detection (LoD), we clinically validated the multiplexed CRISPR-based detection of SARS-CoV-2 *n* and *s* genes (Figure 1B, top) on 136 clinical samples containing a full range of threshold cycle values (13-39). We found the multiplexed CRISPR-based detection of SARS-CoV-2 obtained from nasopharyngeal and throat swabs of infected patients to be 100% specific and 95-97% sensitive compared to RT-qPCR. Within the characterized LoD (C_t_ ~37) the method is 100% specific and 100% sensitive. To allow for broader implementation of the technology during the pandemic, we submitted the most sensitive version of our freeze-dried, multiplexed CRISPR-based detection of SARS-CoV-2 RNA for technological evaluation with the Food and Drug Administration (FDA) of Thailand, and received full approval on September 22, 2021.

With emerging VOCs, we re-designed our multiplexed detection platform—with minimal adjustments to our FDA-approved protocol beyond new primers and crRNAs—to concurrently diagnose SARS-CoV-2 infection—via pan-variant detection of the *n* gene—and discriminate the highly transmissible Delta (B.1.617.2) and Omicron (B.1.1.529) via variant-specific detection of the *s* gene (Figure 1B, bottom). Our best design for multiplexed Delta variant detection showed excellent sensitivity (LoD at C_t_ ~37) and specificity, with no cross-reactivity toward wild-type, Alpha (B.1.1.7), and Omicron SARS-CoV-2. Our Omicron variant detection—which detected the HV69-70 deletion also found in the now extinct Alpha strain—was slightly less sensitive (LoD at C_t_ ~34) and exhibited minimal cross-reactivity with wild-type and Delta SARS-CoV-2.

To sustain SARS-CoV-2 detection and variant surveillance capability in LMICs, we also report here an RT-RPA reaction in which all protein components of RPA were locally produced in Thailand, and demonstrated similar amplification efficiency, including for multiplexed detection, of the locally produced RPA to commercial RPA. Our overall setup (Figure 1C)—an equipment-light, field-deployable, and easy-to-use multiplexed CRISPR-based assay capable of simultaneous detection of SARS-CoV-2 and VOC identification, while maintaining high specificity and sensitivity—can now be prepared with locally manufactured components, providing affordable access of critical reagents for diagnostic and variant surveillance assays to the most vulnerable populations.

## Materials and Methods

### Optimized multiplexed RT-RPA

The optimized reaction condition for multiplexed RT-RPA for the amplification of *s* and *n* genes of SARS-CoV-2 with TwistAmp Basic RPA is as follows. The protocol is for the preparation of 5 multiplexed RPA reactions at a time from one lyophilized RPA pellet (TwistAmp Basic kit, TwistDx). Working multiplexed RPA contained 2.5 μM *s*-gene forward RPA primer, 2.5 μM *s*-gene reverse RPA primer, 3.1 μM *n*-gene F4 forward RPA primer, and 3.1 μM *n*-gene R1 forward RPA primer.

One RPA pellet was resuspended with 29.5 μL of the rehydration buffer from the kit. 1 μL EpiScript reverse transcriptase (200 U/μL stock; Lucigen), 0.36 μL RNase H (5 U/μL stock; NEB), 5 μL triglycine (570 mM stock, Sigma), and 5 μL multiplexed RPA primer mix were added to the RPA resuspension. A portion of 8.2 μL of the RPA-primer-enzyme master-mix was aliquoted into five precooled 1.5 ml Eppendorf tubes. 5.3 μL RNA extract from nasopharyngeal and throat swab samples was then added to each aliquot. Lastly, 0.7 μL magnesium acetate (280 mM stock) was added to initiate the amplification. The reactions were incubated at 42 °C for 25 min, then placed on ice before proceeding to the Cas-based detection step.

### Optimized multiplexed Cas13-based detection with fluorescence readout

Each multiplexed Cas13-based detection reaction can be assembled as follows, and making a mastermix by multiplying the amount given is possible: 2 μL HEPES (200 mM stock, pH 6.8), 0.8 μL ribonucleoside triphosphate mix (rNTPs, 25 mM stock, NEB), 0.6 μL NxGen T7 RNA polymerase (50 U/μL stock, Lucigen), 2.5 μL FAM-polyU-IABkFQ reporter (2 μM stock, IDT), 2.5 μL Cy5-polyA-IABkRQ or ROX-polyA-IABkRQ reporter (4 μM stock, IDT), 1 μL LwaCas13a-crRNA for the *s* gene (10 ng/μL stock, Synthego), 1 μL PsmCas13b-crRNA for the *n* gene (30 ng/μL stock, Synthego), 1 μL LwaCas13a enzyme (126 μg/mL; 900 μM working stock in enzyme storage buffer), 1 μL PsmCas13b enzyme (420 μg/mL; 2700 μM working stock in enzyme storage buffer), 3 μL betaine monohydrate (5 M stock, Sigma), 1 μL magnesium chloride (480 mM stock), and 1.3 μL DEPC-treated water.

Final concentrations of each component were: 20 mM HEPES pH 6.8, 1 mM of each rNTP, 1.5 U/μL T7 RNA polymerase, 250 nM polyU-IABkFQ, 500 nM polyA-IABkRQ, 22.5 nM LwaCas13a crRNA, 67.5 nM PsmCas13b crRNA, 45 nM LwaCas13a enzyme, 135 nM PsmCas13b enzyme, 750 mM betaine monohydrate, and 24 mM magnesium chloride.

After mixing, 18 μL of the multiplexed Cas reaction mixture was transferred to a 384-well-plate well, and 2 μL of the multiplexed RPA reaction was added. Fluorescent signals of FAM, ROX, and Cy5 were monitored using a fluorescence microplate reader (Infinite M Plex, Tecan) at 37 °C. Alternatively, FAM and ROX fluorescence can be visualized using an LED transilluminator (BluPAD Dual LED Blue/White light transilluminator) equipped with plastic lighting gels as excitation and emission filters (LEE Filters, filter no.101 Yellow, 106 Primary red, 119 Dark blue, 139 Primary green, 158 Deep orange).

**Table:** 

Visualization	Light Source	Emission filter	Excitation filter
FAM-ROX	Blue Light	Blue	Orange and yellow
FAM	Blue Light	Blue	Orange and green
ROX	Blue Light	Green	Red
ROX	White Light	Green	Red

**Specific RPA and Cas-based detection methods** for relevant figures, see Table S3 and Table S4.

### Lyophilized RT-RPA for multiplexed gene detection

An RT-RPA reaction buffer A was prepared from 500 μL 50% (w/v) PEG20000, 250 μL of 1 M Tris pH 7.4, and 100 μL of 100 mM DTT. The multiplexed RT-RPA reaction mixture was prepared by resuspending three RPA pellets (TwistAmp Basic kit, TwistDx) with 59 μL of RT-RPA reaction buffer A. 15 μL nuclease-free water, 2 μL EpiScript reverse transcriptase (200 U/μL stock; Lucigen), 0.71 μL RNase H (5 U/μL stock; NEB), 10 μL triglycine (570 mM stock, Sigma), 10 μL of multiplexed RPA primers mix were added to the RPA resuspension. 8.4 μL of the RPA-primer-enzyme master-mix was aliquoted into precooled 1.5 mL Eppendorf tubes; the recipe given was enough to make ten aliquots. The mixture was snap frozen with liquid nitrogen and lyophilized using a freeze dryer (Alpha 2-4 LDplus, Martin Christ) overnight.

To reconstitute and initiate the RT-RPA reaction, the lyophilized RT-RPA pellet was resuspended with 12.4 μL of the RNA sample. Lastly, 1 μL of a solution composed of 196 mM magnesium acetate and 1.5 M potassium acetate was added to each reaction. The reactions were incubated at 42 °C for 25 min, then placed on ice before proceeding to the Cas-based detection using Cas13a-based detection step containing crRNAs for both *s* and *n* genes as previously described. The relative FAM fluorescence intensity was evaluated using a real-time thermal cycler (CFX Connect Real-Time PCR System, Bio-Rad).

### Optimized in-house RT-RPA for single-gene and multiplexed Cas13-based detection

The single-plexed RT-RPA of the *n* gene was set up with the following components (20 μL total reaction volume, consisting of 14.5 μL reagent mastermix and 5.5 μL RNA input): 50 mM Tris (pH 7.5), 100 mM potassium acetate, 14 mM magnesium acetate, 2 mM DTT, 5% (w/v) PEG20000, 200 μM dNTPs, 12 mM ATP, 50 mM phosphocreatine, 100 μg/mL creatine kinase, 150 μg/mL (3.3 μM) UvsX, 30 μg/mL (1.7 μM) UvsY, 900 μg/mL (26.5 μM) Gp32, 120 μg/mL (1.8 μM) Bsu LF, 26.6 U/mL RNase H, 2.8 U/μL EpiScript Reverse Transcriptase, 40 mM triglycine (Sigma), and 700 nM *n* gene primers. 5.5 μL of serially diluted SARS-CoV-2 RNA was used as a template, and the RT-RPA reactions were allowed to proceed at 42°C for 60 min.

The multiplexed RT-RPA to detect the *s* and *n* genes of SARS-CoV-2 was set up in a similar condition as the single-plexed amplification, except for the concentration of specific primers, and the amount of RNA template used. Here, we used the *n* gene and *s* gene RPA primers at 222 nM and 177 nM each, respectively, and add 7 μL of serially diluted SARS-CoV-2 RNA as input. RT-RPA reactions were performed at 42 °C for 60 min, and the RPA products monitored through multiplexed Cas13a/b-based detection as described in the optimized multiplexed Cas13-based detection methods section.

Thereafter, 2 μL of the RPA products were mixed with 18 μL of the Cas13a-based detection reaction, which contained 40 mM Tris-HCl pH 7.4, 60 mM NaCl, 6 mM MgCl_2_, 1 mM of each rNTPs, 1.5 U/μL NxGen T7 RNA polymerase, 6.3 μg/mL LwaCas13a, 1 ng/μL LwaCas13a crRNA (1 ng/μL LwaCas13a crRNA for single gene detection and 0.5 ng/μL LwaCas13a crRNA for *s* gene, 0.5 ng/μL LwaCas13a crRNA for *n* gene for multiplexed detection), and 0.3 μM FAM-PolyU reporter. The generated FAM fluorescence was monitored at 37 °C over 90 min using a real-time thermal cycler (CFX Connect Real-Time PCR System, Bio-Rad).

### Lyophilized in-house RT-RPA for multiplexed gene detection

The multiplexed RT-RPA reaction mixture was prepared by assembling all components as described in the optimized in-house RT-RPA methods section, except that magnesium acetate and potassium acetate were not included, and the mixture was further supplemented with 6% (w/v) trehalose. The mixture was lyophilized using a freeze dryer (Alpha 2-4 LDplus, Martin Christ).

To reconstitute and initiate the RT-RPA reaction, the lyophilized RT-RPA pellet was resuspended in a solution consisting of 1 μL of 280 mM magnesium acetate, 0.4 μL of 5 M potassium acetate, and 17.6 μL of SARS-CoV-2 RNA. The RT-RPA reaction was incubated at 42 °C for 60 min. A portion of the reaction was then transferred to the Cas13a-based detection step containing crRNAs for both *s* and *n* genes as previously described. The generated FAM fluorescence was monitored using a real-time thermal cycler (CFX Connect Real-Time PCR System, Bio-Rad).

### Sample collection, RNA preparation, and ethical approval

As previously described, respiratory samples were collected from patients with suspected SARS-CoV-2 infection at Siriraj Hospital and processed at the Diagnostic Molecular Laboratory, Department of Microbiology, Faculty of Medicine, Siriraj Hospital. Nasopharyngeal and throat swabs were collected in viral transport media (VTM, Gibco). RNA was extracted from 200 μL of the respiratory swabs in VTM using magLEAD® 12gC automated extraction platform (Precision System Science, Japan), eluted in 100 μL buffer, and either used directly for clinical laboratory diagnosis of SARS-CoV-2 via RT-qPCR or stored at -70 °C for later use.

Excess RNA extracts from these samples were then used without any personally identifiable information being collected for our studies. 91 samples with known positive results from RT-PCR for SARS-CoV-2, 45 samples with known negative results from RT-PCR for SARS-CoV-2, 20 Delta clinical samples, 10 Alpha clinical samples, 15 Omicron clinical samples, and 3 samples with positive results for human coronavirus OC43, NL63, and 229E were included for the method validation. Samples were randomized upon giving to study staff, who were kept blinded to the SARS-CoV-2 RT-PCR diagnostics results of the samples when they performed validation experiments of CRISPR diagnostics.

Ethical approval of the study was given by the Siriraj Institutional Review Board (COA: Si 339/2020 and Si 424/2020).

See Supplementary Materials and Methods for cloning methods; protein expression and purification; sequences of RPA primers, crRNAs, and RNA reporters; one-pot multiplexed reactions; in-house RPA optimization experiments; information on cultured SARS-CoV-2 clinical isolates; RT-qPCR and droplet digital RT-PCR; and data analysis and visualization.

## Results

### Developing multiplexed reverse-transcription RPA for SARS-CoV-2 RNA detection

Standard SARS-CoV-2 laboratory diagnostics include the detection of at least two loci, often in a multiplexed manner, as a precaution against escape mutations ^21^; we wished to first confer this multiplexed detection capability to our platform. We focused our optimization of multiplexed isothermal amplification on the RPA technique, as we and others^22^ found another popular isothermal amplification technique, LAMP, to be highly susceptible to carryover contamination, likely due to its generation of large concatemer amplicons that are highly amplificative and resistant to degradation. RPA can also be performed at lower, near ambient temperature, making it more easily deployed for point-of-care or field applications. Our previous work identified four sets of RPA primer pairs which enable amplification of the spike (*s*), nucleocapsid (*n*), and two regions within the replicase polyprotein 1ab (*orf1ab*) genes of SARS-CoV-2^14^ (Figure S1). Their amplification rates were variable, with the primer pair detecting the *s* gene being the most sensitive.^14^ We found that simply combining two of these primer pairs did not result in amplification of both targets, as the fast-amplifying product of one gene would dominate the reaction (Figure S1). In order to achieve simultaneous amplification of two targets, the amplification rates of both targets should match,^23^ and be as high as possible for maximal sensitivity. We decided to focus on improving the amplification of the *n* gene to match that of the *s* gene. The *n* gene has relatively fewer mutations over time^24^ and consistently provides the most sensitive detection in many nucleic acid-based detection assays due to its presence in many subgenomic RNA forms.^25,26^ Screening of 12 RPA primer pair combinations identified a new *n* primer pair (F4-R1) with similar sensitivity of amplification to our *s* gene primers (Figure S1B-C). Combining the optimized *n* and *s* primers in the same reverse-transcription RPA (RT-RPA) reaction containing SARS-CoV-2 RNA as a template allowed us to detect both *n* and *s* amplicons with similar efficiency (Figure S1D). Beyond primer design, we optimized the primer concentration and fine-tuned the *n*:*s* primer pair ratio by increasing the *n* primer pair amount 1.25-1.5-fold, and found the *n* gene detection at these slightly skewed primer pair ratios to improve and match that of the *s* gene at all RNA concentrations tested (Figure S2).

Consistent with previous reports,^14,27^ we found that RNase H improves RT-RPA efficiency, but reducing RNase H amount in RT-RPA 4-8 fold compared to what we initially reported^14^ further enhanced the reaction (Figure S2C). We reasoned that while RNase H can remove RNA from the RNA:DNA duplex product of RT and accelerate subsequent RPA, excess RNase H may degrade the SARS-CoV-2 RNA template in the starting RNA:primer complex and dampen the enzyme’s beneficial effect.

### Optimizing multiplexed CRISPR-based detection for SARS-CoV-2 RNA

We established a readout platform for multiplexed RNA amplification, via a multiplexed CRISPR-Cas reaction. Cas13 enzymes with orthogonal collateral cleavage preferences are well characterized; among the variants, Cas13a from *Leptotrichia wadei* (LwaCas13a) and Cas13b from *Prevotella* sp. MA2016 (PsmCas13b) have already been demonstrated to work well in tandem, and have been used in combination with multiplexed RPA to detect two synthetic DNA targets.^8^ We previously set up LwaCas13a-based detection of SARS-CoV-2 RNA, primarily for the *s* gene, utilizing a FAM-labeled polyU reporter.^14^ To extend the detection to two genes, we expressed and purified PsmCas13b (Figure S3), designed its CRISPR RNA (crRNA) to target the *n* gene of SARS-CoV-2, and prepared a Cy5-labeled polyA reporter to match with adenine cleavage preference of PsmCas13b.

We confirmed that PsmCas13b exhibits collateral cleavage of the polyA reporter, thereby eliciting Cy5 fluorescence when its *n* gene target is present. We empirically optimized the enzyme:crRNA amount used in the detection reaction, as well as the polyA reporter concentration (Figure S4). Generally, more enzyme, crRNA, and the reporter are needed for the PsmCas13b-based detection compared to conditions used for LwaCas13a, reflecting poorer collateral cleavage kinetics of PsmCas13b, which was previously documented^8^ and which we also confirmed by performing side-by-side detection reactions with PsmCas13b vs LwaCas13a on the same RPA product (Figure S5). Despite the slower kinetics of PsmCas13b, we demonstrated that PsmCas13b and LwaCas13a can function orthogonally in a multiplexed reaction, to detect *n* and *s* genes of SARS-CoV-2 respectively (Figure S2D).

We also explored Cas13d from *Ruminococcus flavefaciens* (RfxCas13d), which is the most catalytically active of Cas13 orthologs in mammalian applications,^28^as well as its engineered variant, RfxCas13d-RBD, which has an RNA binding domain (RBD) from human protein kinase R fused to RfxCas13d to improve RNA binding.^29^ However, both RfxCas13d variants exhibited the polyU cleavage efficiency that was lower than that of LwaCas13a (Figure S6). Therefore, we decided to proceed with the current best enzyme pair, LwaCas13a and PsmCas13b, and determine the system’s clinical performance in the multiplexed gene detection of SARS-CoV-2 (Figure S6).

We screened solubility/stability-enhancing additives in the reactions to improve the efficiency of the multiplexed RT-RPA and CRISPR reaction. Triglycine boosted the multiplexed RPA reaction the most, consistently increasing the positive rates (Figure S7A) and betaine monohydrate consistently increased the fluorescence signal generated from both Cas13a- and Cas13b-mediated cleavage by around 2-fold (Figures S7B and S8). The beneficial effect of betaine in the Cas detection step can be combined with that of triglycine in the multiplexed RPA step, and we obtained the best signal-to-noise upon multiplexed detection when both additives were used in their respective reactions. (Figure S7C). Empirical adjustments to the Cas:crRNA ratio and total amount, and reaction buffers further improved the multiplexed Cas reactions (Figure S9).

### Lyophilized RPA and CRISPR-Cas reaction pellets for portable SARS-CoV-2 RNA detection

We simplified liquid handling steps involved in the amplification and detection reactions and increased the shelf-lives of protein/RNA components of the reactions by storing them in lyophilized forms. All components required for either multiplexed RT-RPA or the CRISPR-LwaCas13a-based detection, aside from potassium acetate and magnesium acetate for RT-RPA, and magnesium chloride for CRISPR-Cas reaction, can be lyophilized together in the presence of cryoprotective trehalose with minimal loss of activity upon reconstitution (Figures S10 and S11). Trehalose may help the lyophilized RPA and Cas reactions through two mechanisms: it is known to help stabilize proteins for long-term storage and, from our additive screens, also helps boost the efficiency of both RPA and CRISPR-Cas13 reactions (Figure S7A-B). We kept PEG20000, a well-known cryoprotective agent, in the lyophilized RT-RPA reaction as it functions as a crowding agent for RPA, but PEG20000 adversely affected the CRISPR-Cas reaction upon lyophilization (Figure S11) so was not added to the optimized lyophilized CRISPR-Cas reaction.

Freeze-dried RT-RPA pellet can simply be rehydrated by direct addition of the RNA extract from clinical samples, along with KOAc and Mg(OAc)2. The use of all-in-one freeze-dried RT-RPA pellets has an added benefit in that at least twice the volume of RNA can be used as input, resulting in even higher detection sensitivity compared to our conventional protocol where all reaction components are supplied in liquid form and the RNA input volume is restricted (Figure S11). In addition, increasing the quantity of components in RPA pellets by adding another RPA pellet (RT-3xRPA) boosted the analytical sensitivity of detection in samples with C_t_ as high as 37 (Figure S2E)

### Analysis of clinical specificity, analytical sensitivity (the limit of detection) and clinical sensitivity of the multiplexed detection of SARS-CoV-2 RNA

**Figure 2:**
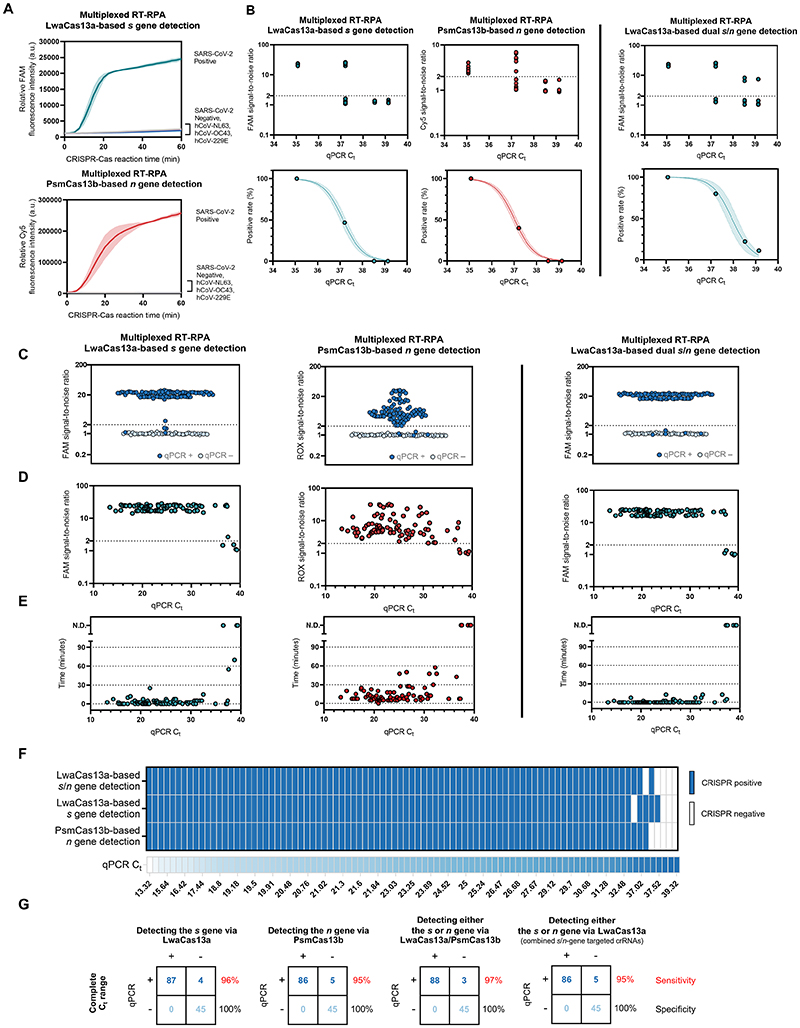
Specificity and sensitivity of the multiplexed CRISPR-based detection of SARS-CoV-2 RNA. **(A)** Clinical specificity. Kinetics of FAM (top) and Cy5 (bottom) fluorescence signal generation from the SARS-CoV-2 *s* (top) and *n* gene (bottom) detection via a multiplexed CRISPR-Cas reaction, using RNA extracts from clinical samples verified to be positive with different coronaviruses via RT-qPCR. The SARS-CoV-2 negative sample is an extract from a clinical sample confirmed to be SARS-CoV-2-negative by RT-qPCR. Data are mean ± s.d. from 3 replicates. (B) The limit of detection. Multiplexed RT-RPA followed by multiplexed Cas-based detection were performed with serially diluted SARS-CoV-2 RNA, whose C_t_ values were determined using a Luna one-step RT-qPCR assay targeting the *n* gene of SARS-CoV-2. At least three independent replicates of the amplification/detection reactions were performed for each SARS-CoV-2 RNA dilution. Top: signal-to-noises (S/N) of FAM (from LwaCas13a-mediated *s* gene detection) and Cy5 (from PsmCas13b-mediated *n* gene detection) fluorescence intensities are shown. The S/N threshold for a positive result was set at 2. Noise is defined as the fluorescence intensity generated from a negative sample with water as input performed in parallel. Bottom: positive rates of the detection of the *s* and *n* genes from multiplexed CRISPR-based detection at different SARS-CoV-2 RNA input concentrations. (C)-(G), clinical sensitivity. (C) Summary of S/N obtained from multiplexed CRISPR-based detection results on 136 clinical samples (91 are COVID-19-positive by RT-qPCR; 45 are negative). (D) Relation of S/N obtained from multiplexed CRISPR-based detection to C_t_ values of COVID-19-positive samples. (E) Time to positive CRISPR detection results vs C_t_ values of COVID-19-positive samples. (F) Results of multiplexed CRISPR-based detection for 91 COVID-19-positive samples ordered by C_t_ values from RT-qPCR. (G) Concordance between RT-qPCR and multiplexed CRISPR-based detection of SARS-CoV-2 RNA. On the right of each concordance box, sensitivity values (%) for the detection of the *s* and *n* genes under a given scheme are shown in red; specificity values (%) are shown in black.

We performed clinical validation with a lyophilized premixed RT-3xRPA formulation, as it had the highest detection sensitivity and suitable features for POC use. We found that the multiplexed RPA and Cas13a/b-based detection are specific for the *s* and *n* genes of SARS-CoV-2, and exhibited no cross-reactivity upon using RNA input from other common human coronaviruses, including human coronavirus OC43 (hCoV-OC43), hCoV-NL63, and hCoV-229E (Figure 2A). Using serially diluted RNA extracts from cultured SARS-CoV-2 in Vero cells (clinical isolate hCoV-19/Thailand/Siriraj_5/2020; GISAID accession ID: EPI_ISL_447908), we determined the limit of detection (LoD) of the optimized multiplexed detection of the SARS-CoV-2 *s* and *n* genes to be at C_t_ ~37 (Figure 2B and Table S5).

The clinical performance of the multiplexed detection platform on RNA extracts from 136 nasopharyngeal and throat swab clinical samples, 91 of which are SARS-CoV-2 positive by RT-qPCR (C_t_ of the *n* gene ranging from ~13-39), matched well with the characterized specificity and LoD (Figure 2C-G). We were able to identify all 45 SARS-CoV-2-negative samples (100% specificity, Figure 2C). Among 91 RT-qPCR-positive samples, we were able to detect both *s* and *n* genes in 85 samples and at least either the *s* or the *n* gene in three other one sample, within 60 min of the multiplexed CRISPR reaction (Figure 2D, E). We were able to detect both genes in samples with C_t_ as high as 37.5 (Figure 2F). The clinical sensitivity of our multiplexed detection within the determined LoD (C_t_ ~37) is 100% for the detection of either the *s* or *n* gene of SARS-CoV-2, and 95% for the detection of both genes (Figure 2G). For the full range of C_t_’s we encountered in clinical samples, the clinical sensitivity is 95-97% for at least one gene detected. Due to extensive optimizations, the limit of detection of our multiplexed detection scheme with lyophilized reagents are even higher than those of freshly prepared singleplex RPA and CRISPR reactions for SARS-CoV-2 *s* gene we previously reported^14^ (LoD C_t_ ~33.5).

In addition to performing multiplexed detection with LwaCas13a and PsmCas13b, we explored the use of LwaCas13a and crRNA combinations to detect both *s*- and *n*-gene RPA amplicons (Figure 2B-G, LwaCas13a-based dual *s*/*n* gene detection). In this scheme, the use of multiple primer pairs targeting different regions of the template could increase the probability of successful amplification by at least one primer pair, especially at very low template concentrations. Specific amplicons generated can all be detected by LwaCas13a—the Cas enzyme with highest collateral activity known to date—programmed with a mixture of crRNAs targeting all desired amplicons. Indeed, the clinical sensitivity for this combined output approach is better than that of the conventional multiplexed detection, reaching 95% for the full C_t_ range in our clinical samples, allowing for highly sensitive detection of SARS-CoV-2 RNA, while maintaining 100% specificity. One notable exception is a clinical sample with C_t_ of ~37.1, which was detected by the Cas13a/Cas13b-based multiplexed detection, but not by the Cas13a-based dual *s/n* gene detection; we attributed this discrepancy to the stochastic nature of detection when analyte concentrations are near the limit of detection. The high level of detection accuracy was maintained upon testing with different SARS-CoV-2 variants (Figure S12). This lyophilized, LwaCas13-based dual-gene detection of SARS-CoV-2 RNA served as a basis for our submission for technological evaluation with the Food and Drug Administration (FDA) of Thailand, who specified precise specificity (no cross-reactivity with Influenza A and B; MERS-CoV; and respiratory syncytial virus) and sensitivity (4,000 SARS-CoV-2 viral copies/mL) requirements for non-PCR molecular tests for SARS-CoV-2. Our test received full approval from the Thai FDA on September 22, 2021.

### A multiplexed CRISPR-based assay for simultaneous universal SARS-CoV-2 detection and SARS-CoV-2 variant differentiation

With suitable RPA primers and crRNA designs incorporating PAM requirements and synthetic mismatches, CRISPR-based detection should have the ability to differentiate single-nucleotide differences^8,9,30–35^ found in pathogen subtypes, including SARS-CoV-2 variants. In practice, cross-detection of highly similar sequences could not be eliminated; this reduced specificity necessitates the use of combinatorial detection of multiple signature mutations to provide high-confidence variant identification.^17^ Here, we sought to be judicious in our target sequence selection and crRNA design such that one CRISPR-Cas reaction is sufficient to identify a SARS-CoV-2 variant. A multiplexed CRISPR-based detection combining a pan-strain detection reaction with a variant differentiation one would be a cost-effective way to simultaneously diagnose COVID-19, and track the emergence and spread of a known VOC in a population.

**Figure 3:**
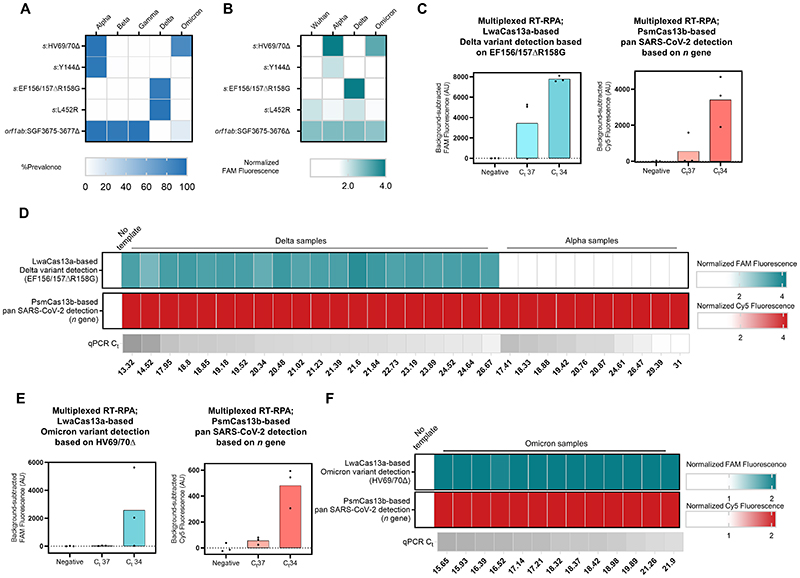
Multiplexed CRISPR-based detection for SARS-CoV-2 variant surveillance (**A**) Prevalence of the target mutations in five major SARS-CoV-2 variants of concern (obtained from https://outbreak.info on , 15 March 2022, Alpha (n = 1,155,468), Beta (n = 41,428), Gamma (n = 120,775), Delta (n = 4,239,814), and Omicron (n = 2,150,574)) (**B**) Selectivity of singleplexed CRISPR-based SARS-CoV-2 variant detection targeting different mutations of interest. FAM fluorescence intensities at 60 minutes were normalized against intensities obtained from the no template control. Data are mean of two replicates. Raw kinetic traces are shown in Figure S16 **(C)** Multiplexed CRISPR-based detection for the Delta variant. The Delta *s* gene was detected via its EF156/157ΔR158G mutation and LwaCas13a. Pan-strain SARS-CoV-2 detection was performed via PsmCas13b-based detection of a conserved region in the *n* gene. Relative FAM (from LwaCas13a-mediated *s* gene detection) and Cy5 (from PsmCas13b-mediated *n* gene detection) fluorescence intensities are shown. Raw kinetic traces are shown in Figure S18A **(D)** Clinical performance of the multiplexed CRISPR-based detection for the Delta variant. Each cell represents a COVID-19-positive clinical sample from Delta (left, n = 20) or Alpha (right, n = 10) lineage. FAM and Cy5 fluorescence intensities at 60 minutes were normalized against intensities obtained from the no template control. **(E)** Multiplexed CRISPR-based detection for the Omicron variant. The Omicron *s* gene was detected via its HV69/70Δ mutation and LwaCas13a. Panstrain SARS-CoV-2 detection was performed via PsmCas13b-based detection of the *n* gene. Relative FAM (from LwaCas13a-mediated *s* gene detection) and Cy5 (from PsmCas13b-mediated *n* gene detection) fluorescence intensities are shown. Raw kinetic traces are shown in Figure S18B **(F)** Clinical performance of the multiplexed CRISPR-based detection for Omicron. FAM and Cy5 fluorescence intensities at 30 minutes were normalized against intensities obtained from the no template control.

In selecting target sequences for variant differentiation, we prioritized indel mutations (range 3-9 base-pairs per mutation) over substitution mutations (a single base-pair per mutation), as the number of bases affected by indel mutations likely result in greater number of mismatches which improves specificity for crRNA discrimination. However, the optimal indel length mismatch between a crRNA and its target is unclear; 1- to 5-nucleotide indels were linked to off-target activation of Cas9,^36^ while a Cas13-based detection has been shown to tolerate up to 15-nucleotide deletion in the target sequence.^8^ While sequence context likely plays a key role, short mismatches may still permit non-cognate crRNA: target recognition, and longer mismatches may allow formation of RNA bulges which minimally interfere with crRNA:target base pairing, also resulting in non-cognate target recognition and Cas activation. We thus decided to empirically test three different indel lengths when we selected SARS-CoV-2 variant-specific mutations: a three-nucleotide deletion (*s*: Y144Δ, specific for Alpha); two six-nucleotide deletions (*s*:HV69/70Δ, specific for Alpha and Omicron BA.1 and BA.3; and *s*:EF156/157ΔR158G; specific for Delta); and a nine-nucleotide deletion (*orf1ab*:SGF3675-3677Δ; specific for Alpha) (Figures 3A and S13). We also tested the detection of one single-nucleotide substitution (*s*:T22917G, resulting in L452R mutation; specific for Delta) and incorporated a synthetic mismatch in our crRNA design (Figure S14).

We designed corresponding RPA primers and LwaCas13a crRNAs for all five mutation-specific CRISPR-based detection (Figures S14 and S15), and first tested their specificity (Figures 3B and S16). The five SARS-CoV-2 variant-specific mutation detection reactions showed SARS-CoV-2 RNA-dependent signal but exhibited different levels of selectivity for their target variants. The two six-nucleotide deletion detections performed the best: EF156/157ΔR158G was highly specific for Delta, with no cross-reactivity with Wuhan, Alpha, or Omicron strains; HV69/70Δ was also highly specific for Alpha and Omicron as designed, but showed low-level cross-reactivity with Wuhan and Delta. Y144Δ, SGF3675-3677Δ and L425R showed clear detection preference for Alpha, Alpha, and Delta respectively, but background signals from other strains were significant. We thus proceeded with the two best-performing variant-specific detection reactions—EF156/157ΔR158G and HV69/70Δ—for subsequent experiments.

We characterized the limit of detection of the singleplex EF156/157ΔR158G-based detection using a dilution series of RNA extracted from cultured SARS-CoV-2 Delta strain, and found the detection to have an excellent LoD, capable of detecting Delta RNA with C_t_ of up to ~38 (Figure S17A). Further evaluation revealed robust detection in clinical samples (C_t_ 15-37) (Figure S17B). We then combined the LwaCas13a-based Delta-specific detection of the *s* gene with the PsmCas13b-based pan-SARS-CoV-2-strain detection of the *n* gene in a multiplexed format (Figure 1B). Using the premixed, lyophilized and multiplexed formulation as previously described, we confirmed that the excellent LoD of Delta-specific reaction was maintained in the multiplexed format (Figures 3C and S18A). Furthermore, the LwaCas13a-based Delta-specific multiplexed detection was able to correctly identify all of twenty Delta patient specimens (C_t_ 13-27), with no cross-reactivity toward ten Alpha clinical specimens (C_t_ 17-31) (Figure 3D). The PsmCas13b-based pan-strain detection was fully functional, detecting the presence of SARS-CoV-2 in this same set of thirty clinical samples.

With the recent emergence of the Omicron variant, we explored the use of the HV69/70Δ-based detection for Omicron identification. We also performed additional cross-reactivity evaluation with higher RNA titer at C_t_ ~25 since we observed sporadic cross-reactivity in the previous experiment (Figures 3B and S19). We found that the HV69/70Δ-based detection was able to distinguish Alpha and Omicron variants from other variants even at higher RNA titer. Next, we demonstrated that the singleplex HV69/70Δ-based detection was sensitive to RNA with C_t_ up to ~34 upon testing with a dilution series of Omicron variant RNA (Figure S17C) and was able to detect all Omicron variant clinical samples (Figure S17D). Using serially diluted RNA from an Omicron clinical extract, we demonstrated sensitive detection of the Omicron variant at up to C_t_ ~34 in a multiplexed format (LwaCas13-based detection of Omicron HV69/70Δ *s* signature, and PsmCas13b-based pan-strain *n* gene detection) (Figures 3E and S18B). In addition, we were able to detect the HV69/70Δ *s* mutation as well as the SARS-CoV-2 *n* gene in fifteen Omicron clinical specimens (C_t_ 16-22), using the HV69/70Δ-based multiplexed detection approach (Figure 3F).

### Combining multiplexed RPA and Cas13-based detection in a single step

**Figure 4:**
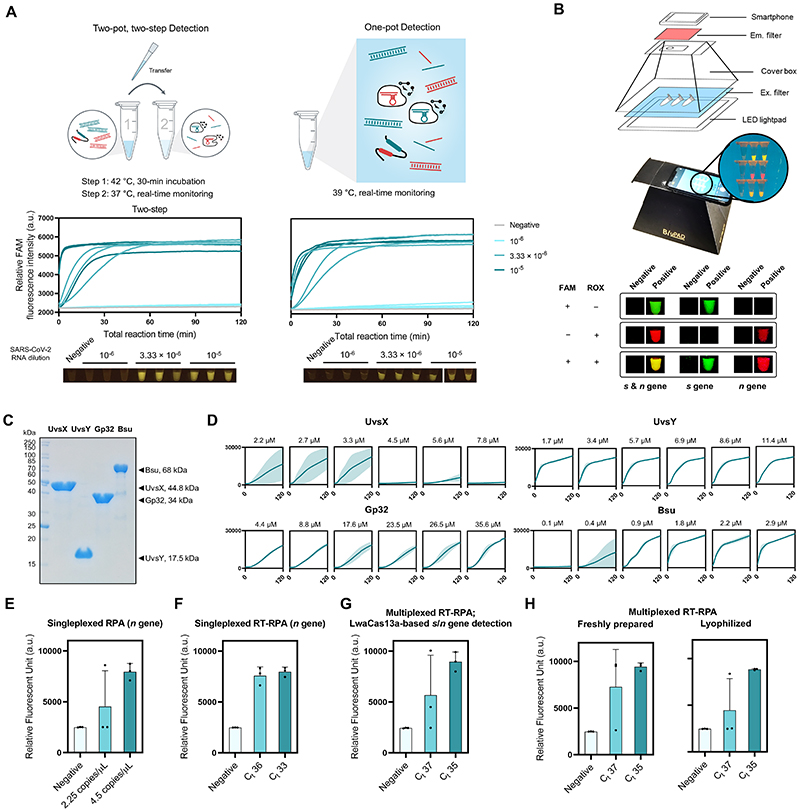
One-pot formulation, easy visualization, and local production of components for RPA and multiplexed CRISPR-based detection. (**A**) The one-pot reaction scheme and its analytical sensitivity compared to the standard two-pot setup. For all, *s*- and *n*-gene amplicons generated from multiplexed RPA were detected with LwaCas13a-based reaction programmed with *s*- and *n*-targeted crRNAs. Data from 3 independent replicates at each RNA input dilution are shown. (**B**) Easy visualization of multiplexed Cas13-based detection. A sample equipment setup with a LED transilluminator, appropriate lighting gels for visualization of fluorescein (FAM) and rhodamine X (ROX), and a smartphone for image capturing is shown. Smartphone-captured images of FAM and ROX signal generated from multiplexed Cas13a/b-based detection of the *s* and *n* genes of SARS-CoV-2. (**C**) SDS-PAGE analysis of locally produced and purified RPA components: UvsX, UvsY, Gp32, and Bsu polymerase large fragment (Bsu LF). (**D**) Optimizations of protein concentrations in in-house RPA. RPA reactions with varying concentrations of UvsX, UvsY, Gp32, and Bsu LF were performed with pUC57-2019-nCoV-N plasmid as a DNA template (at 10,000 copies/μl). Thereafter, the RPA products were used in a LwaCas13a-based detection. FAM fluorescence generated over 120 min for each RPA condition is shown. (**E, F**) Optimally formulated in-house RPA in amplification of the *n* gene of SARS-CoV-2 from pUC57-2019-nCoV-N plasmid (**E**) and serially diluted SARS-CoV-2 RNA (**F**). LwaCas13a-based detection was then used to detect *n*-gene amplicons, with FAM fluorescence intensity generated after 90 min at 37°C shown. (**G**) Optimized in-house RT-RPA for multiplexed detection. Multiplexed RT-RPA to amplify the *s* and *n* genes of SARS-CoV-2 was performed using serially diluted SARS-CoV-2 RNA as a template. Detection of *s* and *n* amplicons was monitored through FAM fluorescence signal generated by Cas13a-mediated *s* and *n* gene detection. The reaction was measured after 90 min at 37°C. (**H**) Lyophilized multiplexed in-house RT-RPA has similar sensitivity as freshly prepared reactions. Amplification and detection of the SARS-CoV-2 *s* and *n* gene are as in (**G**). Data are mean ± s.d. from 3 replicates. RNase-free water was used as input of all negative control reactions.

To simplify usage and minimize the risk of carryover contamination, we formulated a one-pot protocol which combined components of multiplexed RPA and Cas13-based detection in a single reaction tube. We first tested a previously reported one-pot SHINE protocol^37^ for the detection of SARS-CoV-2 *s* gene, but saw that its analytical sensitivity is poorer than our conventional two-step amplification and detection in separate pots by ~10-100-fold. Realizing that concentrations of RPA components in SHINE are diluted by 2-fold compared to the two-step variant, potentially hampering performance of RPA in the one-pot reaction, we re-formulated a one-pot recipe in which final concentrations of all components are as close to those in our optimized two-step variant as possible. We also switched the reaction temperature to be 39 °C, found to be optimal for combined RPA and Cas13-based detection (Figure S21). We tested the optimized one-pot multiplexed RPA amplification of the *s* and *n* genes and concurrent detection by LwaCas13a programmed with *s*- and *n*-targeted crRNAs, and found its analytical sensitivity to be on par with the two-step protocol (Figure 4A).

### Direct visualization of multiplexed Cas13-based detection

To facilitate the use of a multiplexed CRISPR reaction in point-of-care settings, we switched the fluorophore labels of the PsmCas13b-polyA reporter, from Cy5 to rhodamine X (ROX). The ROX-based polyA reporter generates slightly higher levels of signal-to-noise upon PsmCas13b-mediated cleavage compared to the Cy5-based reporter (Figure S22). Moreover, ROX and fluorescein (FAM, on the LwaCas13a-polyU reporter) can be efficiently excited using a single blue LED light source,^38^ under which Cy5 gives no signal. ROX and FAM emissions can then be simultaneously monitored using different combinations of colored plastic filters (Figures 4B, S23 and S24), while maintaining sensitivity of detection upon comparing to microplate reader-based measurements (Figure S25). The emissions are easily readable by eye and ultimately, can be coupled to a portable smartphone-based detection^39–41^ for quantitative analysis.

### Establishing a fully configurable RPA reaction

Given the bottleneck experienced by many labs in procuring and affording commercial RPA, we sought to produce protein components and formulate efficient RPA in house. We expressed the four main protein components of the RPA reaction—bacteriophage T4 recombinase UvsX (UvsX), bacteriophage T4 recombinase loading factor UvsY (UvsY), bacteriophage T4 single-stranded binding protein Gp32 (Gp32), and *Bacillus subtilis* DNA Polymerase I, large fragment (Bsu LF)—in *Escherichia coli* and purified them to about 95% purity (Figure 4C). The activity of our laboratory-made RPA, formulated using a previously reported condition,^42^ was tested via the amplification of a synthetic DNA representing the *s* gene of SARS-CoV-2, which showed clear amplification across different input concentrations under this standard condition (Figure S26). We next titrated the concentrations of the four main protein components of the RPA reaction in order to improve the amplification and found optimal concentrations for each component as follows: 2–3 μM UvsX; 2–11 μM UvsY, 26–35 μM Gp32, and 2-3 μM Bsu LF (Figure 4D). We combined optimized protein concentrations with the triglycine additive to successfully amplify the *n* gene of SARS-CoV-2 from either DNA or RNA samples for subsequent CRISPR-Cas13a detection (Figure 4E-4F). The optimized in-house RT-RPA can be used in a multiplexed amplification reaction of the *s* and *n* genes, coupled to LwaCas13a-based detection with *s*-and *n*-targeted crRNAs (Figure 4G) and can be lyophilized without loss in detection sensitivity (Figure 4H).

## Discussion and outlook

Many advances in point-of-care nucleic acid detection technologies for SARS-CoV-2 RNA—from crude lysate preparation,^13,15,27,37^ nucleic acid enrichment post-lysis to enhance sensitivity,^13,15^ multiple options of isothermal amplification,^43^ amplification-free detection,^12^ multiple modes of detection^14,44^ to mobile, automated readouts of results^12,41^—have collectively pushed toward large-scale practical uses of these technologies for the surveillance of the ongoing pandemic. Here, we extend the utility of CRISPR-based detection—already shown to be among the most sensitive and specific isothermal nucleic acid tests—toward robust multiplexed detection of SARS-CoV-2 RNA. Multi-parameter optimizations of multiplexed RPA and CRISPR-Cas reaction resulted in a highly specific and sensitive detection platform for the *s* and *n* genes of SARS-CoV-2. We critically evaluated its performance on a large set of clinical samples and demonstrated the high sensitivity (95-97%) and specificity (100%) of the multiplexed CRISPR-based detection of SARS-CoV-2 RNA. Our enhanced multiplexed CRISPR-based reaction can be re-designed to simultaneously provide COVID-19 diagnosis and identify the causative SARS-CoV-2 variant of concern, including Delta and Omicron, with high specificity and sensitivity. Beyond variant surveillance, our platform can be used for other multiplexed combinations, including the detection of one SARS-CoV-2 gene and a human gene/RNA control, and the detection of SARS-CoV-2 with another virus, such as influenza, whose infection can co-occur with COVID-19 and present similar symptoms.^45^

Beyond complex detection, we also improve the usability and portability of the enhanced multiplexed CRISPR-based detection platform through freeze-dried reaction mixtures and a one-pot configuration. While the current sensitivity of our enhanced multiplexed platform is already high, we think the key to further improve the test accuracy while maintaining the ease of use of the detection technology lies in the ability to tinker with the RPA reaction itself. The ability to manufacture this key reagent at high quality in the lab has enabled us to make progress toward this goal, first by improving the ease of use through tailored lyophilization of reaction components. In addition to increasing sensitivity, engineering RPA such that the reaction can function directly and robustly in diverse biological fluids could make RPA and CRISPR-based detection viable testing platforms in very limited-resource settings—even at home—for currently circulating or newly emerging SARS-CoV-2 and other infectious agents. Beyond improving technology characteristics, local manufacturing of test kit components creates opportunities for economic and research development, which could lead to sustainable use of these technologies in disease monitoring and diagnosis especially in developing countries.

Both RPA and CRISPR-Cas reactions contain primarily enzyme components; in this study, we started to explore the use of an engineered Cas13 enzyme to improve the reactions. Continual efforts to engineer these enzyme components and their substrates as recently demonstrated with Cas12a ^46^ and its crRNA,^47^ along with integration with automation platforms for high-throughput assay development, could pave way for substantially improved detection platforms.

## Supplementary Material

Supplementary information

## Data Availability

The main data supporting the results in this study are available within the paper and its supplementary information. Raw datasets generated and analysed during the study are available from the corresponding authors upon reasonable request.

## One sentence summary

point-of-care SARS-CoV-2 variant surveillance for LMICs based on multiplexed CRISPR-based detection
