# Supplementary material for: A multiplexed Cas13-based assay with point-of-care attributes for simultaneous COVID-19 diagnosis and variant surveillance
Source: CRISPR J. Author manuscript; Available in PMC 2023 Apr 19. (PMC7614457; doi:10.1089/crispr.2022.0048)
Supplement: Supplementary information [file EMS159661-supplement-Supplementary_information.pdf]

Supplementary Materials

A multiplexed Cas13-based assay with point-of-care attributes for simultaneous COVID-19 diagnosis and variant surveillance

Maturada Patchsung[1,†], Aimorn Homchan[1,2,†], Kanokpol Aphicho[1,†], Surased Suraritdechachai[1,†], Thanyapat Wanitchanon[1,5], Archiraya Pattama[3], Khomkrit Sappakhaw[1], Piyachat Meesawat[1], Thanakrit Wongsatit[1], Artittaya Athipanyasilp[1,3], Krittapas Jantarug[1], Niracha Athipanyasilp[3], Supapat Visanpattanasin[2], Nootaree Niljianskul[4], Pimchai Chaiyen[1], Ruchanok Tinikul[2], Nuanjun Wichukchinda[5], Surakameth Mahasirimongkol[5], Rujipas Sirijatuphat[6], Nasikarn Angkasekwinai[6], Michael A. Crone[7,8,9], Paul S. Freemont[7,8,9], Julia Joung[10,11,12,13,14], Alim Ladha[10,11,12,13,14], Omar Abudayyeh[12], Jonathan Gootenberg[12], Feng Zhang[10,11,12,13,14], Claire Chewapreecha[15,16], Sittinan Chanarat[2], Navin Horthongkham[3,*], Danaya Pakotiprapha[2,*], Chayasith Uttamapinant[1,*]

[*]Corresponding authors: danaya.pak@mahidol.ac.th; navin.hor@mahidol.edu; chayasith.u@vistec.ac.th

Supplementary Materials and Methods

Cloning methods for RPA expression plasmids

Nucleic acid sequences codon optimized for *E. coli* expression for T4 UvsX, T4 UvsY, T4 gp32, and Bsu DNA polymerase large fragment (Bsu LF) were ordered as plasmids from GeneArt (ThermoFisher Scientific). Bsu LF and *uvsY* were cloned into the pET28a backbone using Circular Polymerase Extension Cloning (CPEC).[1] *Gp32* and *uvsX* were cloned into the pET28a backbone using Type IIS assembly.[2] Plasmids were sequence verified (Eurofins Genomics) and are available on Addgene as follows: pET28a-MH6-Bsu LF (Plasmid #163911); pET28a-gp32-H6 (Plasmid #163912); pET28a-uvsX-H6 (Plasmid #163913); and pET28a-MH6-uvsY (Plasmid #163914).

Expression and purification of protein components of RPA

We used near-identical expression and cell lysis protocols for UvsX, UvsY, gp32, and Bsu LF, but the purification step and final storage buffer conditions were different for each enzyme, as given below.

An expression plasmid for UvsX, UvsY, gp32, or Bsu LF was transformed into *E. coli* BL21(DE3) cells. The cells were grown in LB medium at 37°C until OD_{600} reached 0.7–0.8. Protein expression was induced using the following concentrations of isopropyl-β-D-thiogalactopyranoside (IPTG): 1 mM for UvsX and UvsY; 0.2 mM for gp32; and 0.5 mM for Bsu LF. The cells were grown for additional 16 hours at 16°C and harvested by centrifugation. The cell pellet was resuspended in lysis buffer (50 mM sodium phosphate pH 8.0, 500 mM sodium chloride, 10 mM imidazole). Phenylmethylsulfonyl fluoride (PMSF) was added into resuspended solution at 1 mM final concentration followed by sonication for a total burst time of 3 minutes (3 sec on for short burst and 9 sec off for cooling). The cell lysate was clarified by centrifugation at 27,000×g for 30 minutes at 4 °C. Nickel-nitrilotriacetic acid (Ni-NTA) agarose beads (Qiagen) was washed with two column volumes of water and equilibrated with lysis buffer. The clarified cell lysate was incubated with the Ni-NTA agarose beads (Qiagen) at 4 °C for 40 minutes. The column was washed with ten column volumes (CVs) of washing buffer (50 mM sodium phosphate pH 8.0, 500 mM sodium chloride, and 20 mM imidazole), and the bound proteins were eluted with elution buffer (50 mM sodium phosphate pH 8.0, 500 mM sodium chloride, 250 mM imidazole).

For UvsX, the protein was further purified by Heparin Sepharose Fast Flow (GE Healthcare Life Sciences) with a linear gradient of 0.1–1 M NaCl in Buffer A (20 mM Tris-HCl pH 8, 5 mM β-mercaptoethanol (βME)). Proteins were concentrated and stored in 20 mM Tris-HCl pH 8.0, 500 mM NaCl, and 40% (v/v) glycerol at −20 °C.

For UvsY, the protein was further purified by Heparin Sepharose Fast Flow (GE Healthcare Life Sciences) with a linear gradient of 0.1–1 M NaCl in Buffer A (20 mM Tris-HCl pH 8, 5 mM βME). Afterward, proteins were dialyzed against 20 mM Tris-HCl pH 7.5, 400 mM NaCl, 20% (v/v) glycerol and 5 mM βME, and the glycerol concentration adjusted to be 50% (v/v) with the addition of UvsY freezing buffer (20 mM Tris-HCl pH 7.5, 400 mM NaCl, and 80% (v/v) glycerol). Protein aliquots were stored in 20 mM Tris-HCl pH 7.5, 400 mM NaCl, and 50% (v/v) glycerol at −20 °C.

For gp32, eluted protein fractions were dialyzed against 20 mM Tris-HCl pH 7.5, 400 mM NaCl, and 5 mM βME, and the glycerol concentration adjusted to be 50% (v/v). Protein aliquots were stored in 10 mM Tris-HCl pH 7.5, 200 mM NaCl, and 50% (v/v) glycerol at −20 °C.

For Bsu LF, eluted protein fractions were concentrated and dialyzed against 50 mM Tris-HCl pH 7.5, 50 mM KCl, 1 mM DTT, 0.1 mM EDTA, and 20% (v/v) glycerol. Glycerol concentration was adjusted to be 50% (v/v) with the addition of Bsu LF freezing buffer (50 mM Tris-HCl pH 7.5, 50 mM KCl, 1 mM DTT, 0.1 mM EDTA, and 90% (v/v) glycerol). Protein aliquots were stored in 50 mM Tris-HCl pH 7.5, 50 mM KCl, 1 mM DTT, 0.1 mM EDTA, and 50% (v/v) glycerol at −20 °C.

Expression and purification of Cas enzymes

LwaCas13a expression and purification

LwaCas13a was expressed from pC013-His_6-Twinstrep-SUMO-LwaCas13a (Addgene: #90097) and purified as previously described[3].

PsmCas13b expression and purification

Escherichia coli BL21 (DE3) transformed with pC0061-His_6-Twinstrep-SUMO-PsmCas13b (Addgene: #115211) were grown in LB media containing 25 μg/mL ampicillin, and the protein expression induced by the addition of 500 μM IPTG at 16 °C for overnight. Cells were collected by centrifugation at 8,000 rpm for 20 min at 4 °C and the supernatant was discarded. The cell pellet was resuspended in extraction buffer (50 mM Tris-HCl, 500 mM NaCl, 1 mM DTT, 1x protease inhibitor cocktail, 0.25 mg/mL lysozyme, 5 mM imidazole, pH 7.5) then lysed by sonication (Sonics Vibracell VCX750) using 40-60% pulse amplitude (on 10 s and off 20 s until completely lysed). The lysate was centrifuged at 15,000 rpm for 45 min at 4 °C, and the soluble fraction filtered through a 0.45 μm polyethersulfone membrane and loaded into Chelating Sepharose™ Fast Flow column which was pre-loaded with 0.2 M $NiSO_4$ and pre-equilibrated with binding buffer (50 Tris-HCl, 500 mM NaCl, 5 mM imidazole pH 7.5). The column was washed with 5 column volumes of binding buffer, 6 column volumes of washing buffer (50 Tris-HCl, 500 mM NaCl, 50 mM imidazole pH 7.5) and eluted with 6 column volumes of elution buffer (50 Tris-HCl, 500 mM NaCl, 500 mM imidazole pH 7.5). The fractions containing His-Twinstrep-SUMO-PsmCas13b were pooled and exchanged with SUMO cleavage buffer (20 mM Tris-HCl, 150 mM NaCl, pH 8.0) at 4 °C overnight.

The SUMO tag was cleaved with Ulp1 SUMO protease (Addgene #64697) using 10:1 of SUMO substrate by incubation at room temperature for 5 h. The reaction was adjusted to pH 6.0 and filtered with 0.45 μm polyethersulfone membrane before loading into HiTrap SP HP column which was equilibrated with binding buffer (50 mM phosphate pH 6.0, 200 mM NaCl). The protein was washed and eluted with 50 mM phosphate, 200 mM NaCl with pH ranging from 6.0, 6.5, 7.0, 7.5, and 8.0, followed by 50 mM Tris pH 8.0, 1 M NaCl. The PsmCas13b fractions were pooled, concentrated, exchanged buffer using centricon 30k, diluted to a concentration of 1.2 mg/mL, and stored at -80 °C in storage buffer (50 mM Tris-HCl, 600 mM NaCl, 5 % glycerol, 2 mM DTT).

RfxCas13d and RfxCas13d-RBD expression and purification

E. coli BL21 (*DE3*) cells transformed with CMS1371 (for His_6-MBP-RfxCas13d expression) or CMS1372 (for His_6-MBP-RfxCas13d-dsRBD) plasmid were grown in LB media containing 100

µg/mL ampicillin, and the recombinant gene expression induced by the addition of 500 µM IPTG at 16 ºC for overnight. Cells were collected and lysed as for PsmCas13b. Following lysate clarification, the soluble fraction was filtered through 0.2 µm polyethersulfone membrane and purified by HisTrap FF column connected to FPLC system. The column was pre-equilibrated with binding buffer (50 mM Tris-HCl, 500 mM NaCl, 5 mM imidazole; pH 7.5). The soluble fraction was loaded at 2 mL/min, washed with 5 column volumes of binding buffer. The recombinant protein was eluted in linear gradient of elution buffer (50 mM Tris-HCl, 0.5 M NaCl, and 0.5 M imidazole; pH 7.5). The eluted fractions were pooled and dialyzed with 50 mM Tris-HCl, 0.5 mM EDTA, 1 mM DTT and 200 mM NaCl; pH 7.5 at 4 ºC for 3 hours, before proceeding to MBP tag removal.

The MBP tag was cleaved with ultraTEV protease using 20:1 substrate: protease ratio in 50 mM Tris-HCl, 0.5 mM EDTA, 1 mM DTT and 200 mM NaCl; pH 7.5. The reaction mixture was incubated at 4 ºC for overnight with gentle shaking before applied onto HisTrap column, which was pre-equilibrated with (50 mM Tris-HCl, 500 mM NaCl, 5 mM imidazole; pH 7.5). The flow-through and unbound fractions containing RfxCas13d and RfxCas13d-dsRBD protein were then collected while retained MBP and other contaminants were later washed out with high concentration of imidazole. The purified protein was exchanged against 40 mM Tris, 400 mM NaCl; pH 7.5 in DEPC-treated water and concentrated using centricon 30k, and diluted to a concentration of 1.22 mg/mL (for RfxCas13d) and 1.89 mg/mL (for RfxCas13d-RBD).

RPA primers, crRNAs, and RNA reporters

The oligonucleotides used are listed in Table S2. The crRNAs used for variant detection were synthesized via *in vitro* transcription. The T7-3G oligonucleotide (at the final concentration of 0.5 µM) and the ssDNA crRNA template (at the final concentration of 0.5 µM) were subjected to 34 cycles of 50 µL Q5® High-Fidelity DNA Polymerase reaction (New England Biolabs). PCR products were purified using DNA Clean & Concentrator-5 kits (Zymo Research) and eluted in 20 µL of nuclease-free water. Four picomoles of the purified dsDNA crRNA template were used in *in vitro* transcription reactions using RiboMAX Large Scale RNA Production System–T7 (ProMega) or MEGAshortscript™ T7 Transcription Kit (Invitrogen). The reactions were performed at 37 ºC overnight, treated with DNase I, and purified using phenol-chloroform extraction and alcohol precipitation. 15% acrylamide/7.5 M urea PAGE with GelRed® (Biotium) staining was used to assess the size and purity of the transcribed product. Gels were imaged on ImageQuant™ LAS 4000 (GE Healthcare); quantifications of produced crRNAs were performed by measuring band densitometries using Fiji ImageJ software[4] and comparing to RNA standards.

Design of RPA primers and crRNAs

General RPA primers and crRNAs design

RPA primers and crRNA were designed according to a published protocol.[5]

Design of RPA primers and crRNA targeting specific SARS-CoV-2 mutations

Representative genome sequences of Alpha (n = 1,100), Beta (n = 126), Gamma (n = 729), and Delta (n = 1,329) were retrieved from NCBI SARS-CoV-2 data packages using PANGO designations[6] as queries (dates of data retrieval, 27 April and 5 May 2021). Retrieved sequences were aligned using MAFFT[7]. The alignments were visualized and used to create consensus sequence for each variant using Jalview.[8] The four consensus sequences along with the SARS-

CoV-2 isolate Wuhan-Hu-1 NCBI reference genome (Accession ID NC_045512.2) were re-aligned using MAFFT and visualized in SnapGene software (Insightful Science). Using visualized multiple sequence alignment, RPA primers and crRNAs were manually designed to cover regions spanning the target mutation while avoiding regions with genetic variations among SARS-CoV-2 variants. The design and chosen regions are shown in Figure S14 and Figure S15.

Exclusivity evaluation of designed crRNAs
crRNA spacer sequences and their complementary sequences were searched against a BLAST database of Betacoronavirus nucleotide sequences (Betacoronavirus BLAST, https://blast.ncbi.nlm.nih.gov/Blast.cgi?PAGE_TYPE=BlastSearch&BLAST_SPEC=Betacoronavirus), with the maximal number of target sequences set to 5000. The results were filtered to include complete genomes (>29,000 nt) with 90-100% query coverage and 90-100% identity (date of data retrieval 1 March 2022). The clade assignment of genomes was done using NextClade (https://clades.nextstrain.org)[9]. The genomes with bad and mediocre NextClade overall quality control status were excluded. The analysis was summarized in Figure S20.

Inclusivity evaluation of designed primers and crRNAs
GenBank accession lists of SARS-CoV-2 variant genomes sampling by the NextStrain COVID-19 global analysis open-data build were acquired by applying filter data by clade before downloading author metadata (date of data retrieval, 3 March 2022); Alpha (n = 110), Beta (n = 30), Gamma (n = 31), Delta (n = 1,044), and Omicron (n = 785)). FASTA files comprising listed genomes were downloaded from GenBank and aligned using MAFFT. The resulted alignments were visualized and inspected using JalView[9]. The prevalence of the sequences aligned with primers and crRNAs in different variants were calculated as a percentage of genomes with the complete matched sequence observed in the alignments and represented by the frequency of the sequence among the variant isolates (%F). Consensus sequences of each variant along with their %F values are provided in Figure S15 for primers and Figure S14 for crRNAs.

The NextStrain Clades or PANGO lineages were designated to each WHO classified variant of concern as described in Table S1

RPA Primer and primer combination screening with colorimetric lateral-flow Cas13a-based readout (for **Figure S1**)
RT-RPA was set up as previously described[3] using TwistAmp Basic Kit (TwistDx) and EpiScript reverse transcriptase (Lucigen). Sequences of RPA primers used were given in Table S2. LwaCas13a-based detection reactions were also set up exactly as previously described,[3] then visualized with HybriDetect lateral-flow strips (Milenia Biotec). Sequences of LwaCas13a-crRNAs for each amplicon were given in Table S2.

One-pot, multiplexed RT-RPA/CRISPR-Cas13a detection
We prepare the one-pot, monophasic detection reactions by preparing the multiplex RT-RPA and CRISPR-Cas13a reaction mixes separately, before mixing them together, as follows.

RT-RPA reaction buffer and multiplexed RPA primers mix were prepared as described above. crRNA-reporter solution was prepared from 24 µL DEPC-treated water, 4 µL of 100 ng/µL LwaCas13a crRNA for *s* gene, 4 µL of 100 ng/µL LwaCas13a crRNA for *n* gene, and 2 µL of 100

μM FAM-PolyU reporter. Then, CRISPR-Cas13a reaction mix was prepared by combining 2.8 μL rNTPs mix (25 mM each), 2 μL T7 RNA polymerase (50 U/μL), 7 μL LwaCas13a (63 μg/mL), and 3 μL of the crRNA-reporter solution.

The multiplexed RT-RPA reaction mixture was prepared by resuspending one RPA pellet (TwistAmp Basic kit, TwistDx) with 14.2 μL of RT-RPA reaction buffer A. 1 μL EpiScript reverse transcriptase (200 U/μL stock; Lucigen), 0.36 μL RNase H (5 U/μL stock; NEB), 5 μL triglycine (570 mM stock, Sigma), 5 μL of multiplexed RPA primers mix, and 15 μL of CRISPR-Cas13a reaction mix were then added to the RPA resuspension. 8.2 μL of the RPA-primer-enzyme master-mix was aliquoted into precooled 0.1 mL PCR strip tubes; the recipe given was enough to make 5 aliquots.

To initiate amplification and detection, 5.3 μL of the RNA sample was added to each aliquot, followed by 0.7 μL magnesium acetate (280 mM). The reactions were incubated at 39 °C and generated FAM fluorescence was monitored using a real-time thermal cycler (CFX Connect Real-Time PCR System - Bio-Rad).

Standard (unoptimized) in-house RPA reaction

Standard RPA reactions were set up as previously reported[10] and contained the following components: 50 mM Tris (pH 7.5), 100 mM potassium acetate, 14 mM magnesium acetate, 2 mM DTT, 5% (w/v) PEG20000, 200 μM dNTPs, 3 mM ATP, 50 mM phosphocreatine, 100 μg/mL creatine kinase, 120 μg/mL UvsX, 30 μg/mL UvsY, 900 μg/mL Gp32, 30 μg/mL Bsu LF, 450 nM primers, and DNA templates. Reactions were performed in 20 μL volumes at 37 °C for 60 min, followed by inactivation at 65 °C for 10 min. Ten microliters of each reaction were mixed with 2% (w/v) final concentration of sodium dodecyl sulfate (SDS) and Novel Juice DNA staining reagent (BIO-HELIX), separated on 2% (w/v) agarose gel and visualized under blue light (BluPAD, BIO-HELIX).

Optimizing RPA through titration of protein components

Concentrations of the main protein components of RPA (UvsX, UvsY, Gp32, and Bsu LF) were varied as indicated in Figure 4D. We also switched to using RPA primers for the *n* gene amplification (the F4/R1 pair) and pUC57-2019-nCoV-N plasmid (MolecularCloud cat no. #MC_0101085) at 10,000 copies/μL as the DNA template. The RPA reaction conditions were otherwise identical to the standard RPA reaction (see previous section), and were allowed to proceed at 42°C for 60 min. Thereafter, 2 μL of the RPA products were mixed with 18 μL of the Cas13a-based detection reaction, which contained 20 mM Tris-HCl pH 7.4, 60 mM NaCl, 6 mM $MgCl_2$, 1 mM of each rNTPs, 1.5 U/μL NxGen T7 RNA polymerase, 6.3 μg/mL LwaCas13a, 0.5 ng/μL LwaCas13a crRNA, and 0.3 μM FAM-PolyU reporter. The generated FAM fluorescence was monitored at 37 °C over 90 min using a fluorescence microplate reader (Varioskan, Thermo Scientific or Infinite M Plex, Tecan).

Optimized in-house RPA for single-gene detection

The single-plexed RPA of the *n* gene was set up with the following components (20 µl total reaction volume, consisting of 19 μL reagent mastermix and 1 μL RNA input): 50 mM Tris (pH 7.5), 100 mM potassium acetate, 14 mM magnesium acetate, 2 mM DTT, 5% (w/v) PEG20000, 200 μM dNTPs, 12 mM ATP, 50 mM phosphocreatine, 100 μg/mL creatine kinase, 150 μg/mL

(3.3 μM) UvsX, 30 μg/mL (1.7 μM) UvsY, 900 μg/mL (26.5 μM) Gp32, 120 μg/mL (1.8 μM) Bsu LF, 40 mM triglycine (Sigma), and 700 nM *n* gene primers. One μL of pUC57-2019-nCoV-N plasmid (MolecularCloud cat no. #MC_0101085) was used as a template, and the RPA reactions were allowed to proceed at 42°C for 60 min. Thereafter, 2 μL of the RPA products were mixed with 18 μL of the Cas13a-based detection reaction, which contained 40 mM Tris-HCl pH 7.4, 60 mM NaCl, 6 mM $MgCl_2$, 1 mM of each rNTPs, 1.5 U/μL NxGen T7 RNA polymerase, 6.3 μg/mL LwaCas13a, 1 ng/μL LwaCas13a crRNA for *n* gene, and 0.3 μM FAM-PolyU reporter. The generated FAM fluorescence was monitored at 37 °C over 90 min using a real-time thermal cycler (CFX Connect Real-Time PCR System, Bio-Rad).

Cultured SARS-CoV-2 viral RNA extracts and serial dilutions

SARS-CoV-2 Wuhan variant (clinical isolate hCoV-19/Thailand/Siriraj_5/2020; GISAID accession ID: EPI_ISL_447908), Alpha variant (clinical isolate hCoV-19/Thailand/Bangkok_R098/2021; GISAID accession ID: EPI_ISL_11000098), and Delta variant (clinical isolate hCoV-19/Thailand/Bangkok_SEQ4389/2021; GISAID accession ID: EPI_ISL_3038904) were propagated in Vero E6 cells cultured in MEM-E supplemented with 10% fetal bovine serum. The culture media were collected and extracted as described for the clinical sample. The RNA extracts at given dilution were prepared by dilution with nuclease-free water and stored at -70 °C for later use.

RT-qPCR

The RT-qPCR was performed according to a published protocol.[11] In brief, a 5 μL of the RNA sample was added to a 20-μL reaction prepared with Luna® Universal Probe One-Step RT-qPCR Kit (New England Biolabs) and nCoV_N1 primer-probe set (Integrated DNA Technologies). The reactions were monitored using CFX Connect Real-Time PCR System (Bio-Rad).

Allplex™ SARS-CoV-2 Variants I Assay (Seegene, Korea), which targets *RdRp* and *S*; delH69/V70, E484K and N501Y of SARS-CoV-2, was used for SARS-CoV-2 Alpha and Omicron variant screening. Allplex™ SARS-CoV-2 Variants II Assay (Seegene, Korea), which targets *RdRp* and *S*; K417N, K417T, L452R and W152C, was used for Delta variant screening. All identified variant samples were confirmed with full length spike sequencing.

Droplet digital RT-PCR (RT-ddPCR)

The RT-ddPCR was performed using One-Step RT-ddPCR Advanced Kit for Probes (Bio-Rad) according to the manufacturer's protocol and in line with RT-qPCR protocol described above. A 20-μL reaction consisted of 5 μL of RNA input, 5 μL of Supermix, 2 μL of reverse transcriptase, 1 μL of 300 mM DTT, 4 μL nuclease-free water, and each 1 μL of following component from nCoV_N1 primer-probe set (Integrated DNA Technologies): 10 μM forward primer, 10 μM reverse primer, and 10 μM probe. The reaction droplets were generated and read using a QX100™ Droplet Digital™ PCR system (Bio-Rad) connected to a T100™ Thermal Cycler (Bio-Rad). Thermocycling conditions were reverse transcription at 50 °C for 60 minutes, enzyme activation at 95 °C for 10 minutes, followed by 40 cycles of 95 °C for 15 seconds, 55 °C for 30 seconds, 58 °C for 10 seconds. The enzyme deactivation was done at 98 °C for 10 minutes. The quantification was carried out using QuantaSoft™ software (Bio-rad).

Standard curve of qPCR Ct versus ddPCR-derived copies/uL RNA input see Figure S27

Data analysis and visualization

Statistical analyses were performed, and graphical representations created with GraphPad Prism 9, unless indicated otherwise. Schematic diagrams were created with Biorender.com and Adobe Illustrator CC 2017.

Supplementary references

1. Quan J and Tian J. Circular Polymerase Extension Cloning for High-Throughput Cloning of Complex and Combinatorial DNA Libraries. Nature Protocols 2011;6(2):242–251; doi: 10.1038/nprot.2010.181.
2. Engler C, Kandzia R and Marillonnet S. A One Pot, One Step, Precision Cloning Method with High Throughput Capability. El-Shemy HA. ed. PLoS ONE 2008;3(11):e3647; doi: 10.1371/journal.pone.0003647.
3. Patchsung M, Jantarug K, Pattama A, et al. Clinical Validation of a Cas13-Based Assay for the Detection of SARS-CoV-2 RNA. Nature Biomedical Engineering 2020;4(12):1140–1149; doi: 10.1038/s41551-020-00603-x.
4. Schindelin J, Arganda-Carreras I, Frise E, et al. Fiji: An Open-Source Platform for Biological-Image Analysis. Nature Methods 2012;9(7):676–682; doi: 10.1038/nmeth.2019.
5. Kellner MJ, Koob JG, Gootenberg JS, et al. SHERLOCK: Nucleic Acid Detection with CRISPR Nucleases. Nature Protocols 2019;14(10):2986–3012; doi: 10.1038/s41596-019-0210-2.
6. Rambaut A, Holmes EC, O'Toole Á, et al. A Dynamic Nomenclature Proposal for SARS-CoV-2 Lineages to Assist Genomic Epidemiology. Nature Microbiology 2020;5(11):1403–1407; doi: 10.1038/s41564-020-0770-5.
7. Katoh K and Standley DM. MAFFT Multiple Sequence Alignment Software Version 7: Improvements in Performance and Usability. Molecular Biology and Evolution 2013;30(4):772–780; doi: 10.1093/molbev/mst010.
8. Waterhouse AM, Procter JB, Martin DMA, et al. Jalview Version 2-A Multiple Sequence Alignment Editor and Analysis Workbench. Bioinformatics 2009;25(9):1189–1191; doi: 10.1093/bioinformatics/btp033.
9. Aksamentov I, Roemer C, Hodcroft E, et al. Nextclade: Clade Assignment, Mutation Calling and Quality Control for Viral Genomes. Journal of Open Source Software 2021;6(67):3773; doi: 10.21105/joss.03773.
10. Piepenburg O, Williams CH, Stemple DL, et al. DNA Detection Using Recombination Proteins. PLoS Biology 2006;4(7):1115–1121; doi: 10.1371/journal.pbio.0040204.
11. Vogels CBF, Brito AF, Wyllie AL, et al. Analytical Sensitivity and Efficiency Comparisons of SARS-CoV-2 RT–QPCR Primer–Probe Sets. Nature Microbiology 2020;5(10):1299–1305; doi: 10.1038/s41564-020-0761-6.

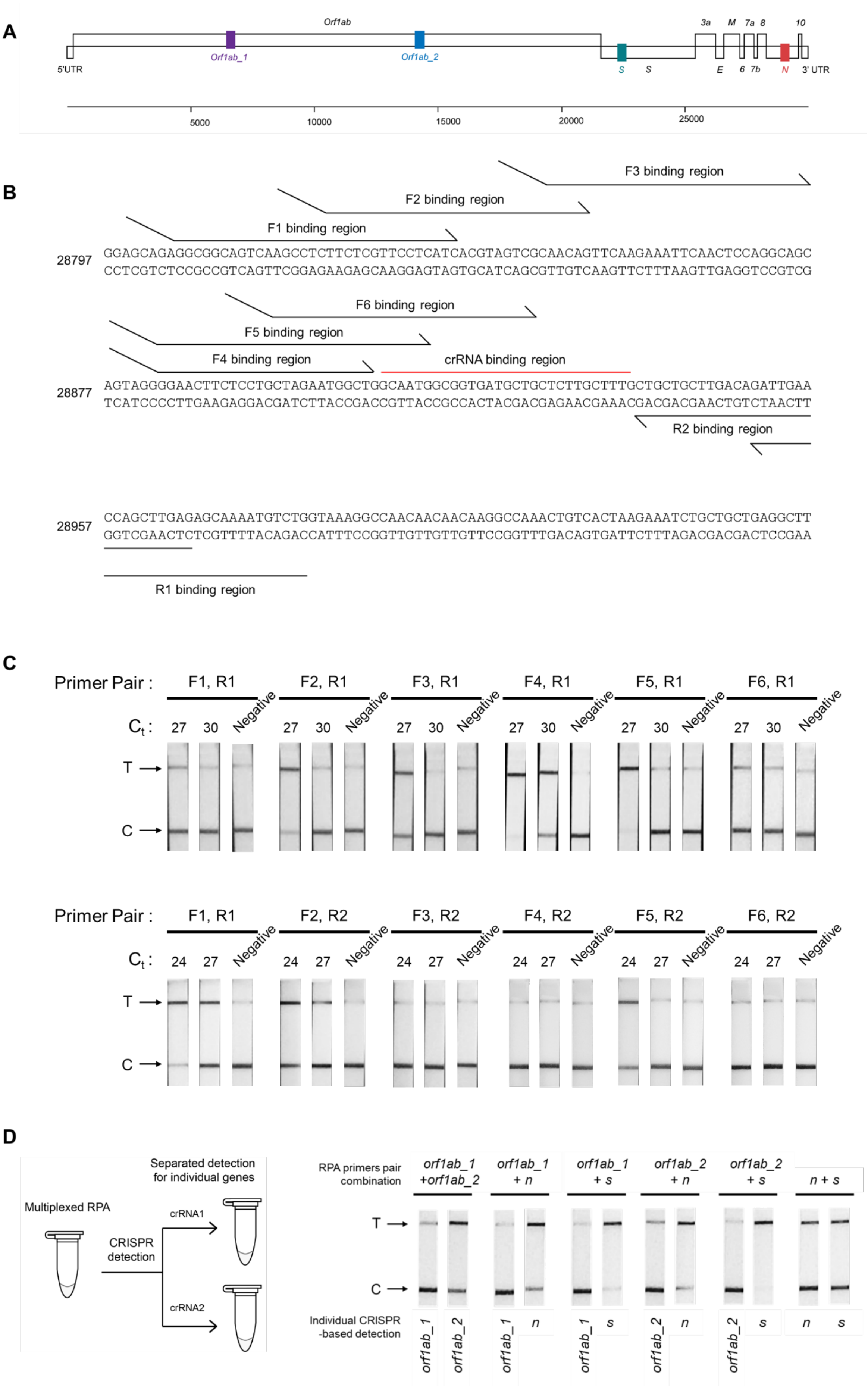
A
Orf1ab
Orf1ab_1
Orf1ab_2
5'UTR
S
S
3a
M
7a
8
10
E
6
7b
N
3' UTR
5000
10000
15000
20000
25000
B
F3 binding region
F2 binding region
F1 binding region
28797
GGAGCAGAGGCGGCAGTCAAGCCTCTTCTCGTTCCTCATCACGTAGTCGCAACAGTTCAAGAAATTCAACTCCAGGCAGC
CCTCGTCTCCGCCGTCAGTTCGGAGAAGAGCAAGGAGTAGTGCATCAGCGTTGTCAAGTTCTTTAAGTTGAGGTCCGTCG
F6 binding region
F5 binding region
F4 binding region
crRNA binding region
28877
AGTAGGGGAACTTCTCCTGCTAGAATGGCTGGCAATGGCGGTGATGCTGCTCTTGCTTTGCTGCTGCTTGACAGATTGAA
TCATCCCCTTGAAGAGGACGATCTTACCGACCGTTACCGCCACTACGACGAGAACGAAACGACGACGAACTGTCTAACTT
R2 binding region
28957
CCAGCTTGAGAGCAAAATGTCTGGTAAAGGCCAACAACAACAAGGCCAAACTGTCACTAAGAAATCTGCTGCTGAGGCTT
GGTCGAACTCTCGTTTTACAGACCATTTCCGGTTGTTGTTGTTCCGGTTTGACAGTGATTCTTTAGACGACGACTCCGAA
R1 binding region
C
Primer Pair :
F1, R1
F2, R1
F3, R1
F4, R1
F5, R1
F6, R1
Ct :
27
30
Negative
T
C
Primer Pair :
F1, R1
F2, R2
F3, R2
F4, R2
F5, R2
F6, R2
Ct :
24
27
Negative
T
C
D
Separated detection for individual genes
Multiplexed RPA
crRNA1
CRISPR detection
crRNA2
RPA primers pair combination
orf1ab_1 +orf1ab_2
orf1ab_1 + n
orf1ab_1 + s
orf1ab_2 + n
orf1ab_2 + s
n + s
T
C
Individual CRISPR -based detection
orf1ab_1
orf1ab_2
n
s

Figure S1: **Optimizing the RT-RPA amplification of SARS-CoV-2 *n* gene.**
(**A**) Four regions within the *orf1ab*, *s*, and *n* genes of SARS-CoV-2 genome selected for detection are highlighted. (**B**) Binding positions of different primers to the SARS-CoV-2 *n* gene. (**C**) Testing efficiency of different primer pairs on SARS-CoV-2 RNA. RT-RPA using each primer pair was performed with SARS-CoV-2 RNA at two different concentrations as a template. Cas13a-based detection with lateral-flow readout was then used to assess successful amplification. Experiments with R1 reverse primers (Qian et al.) and R2 reverse primers (bottom) were performed using different SARS-CoV-2 RNA dilutions, but with an identical control primer pair (F1, R1) included in both sets of experiments, allowing us to compare relative performance of all primer pairs. Based on this screening, we selected the (F4, R1) primer pair for subsequent *n* gene amplifications. **D**) Multiplexed RPA reactions with pairwise combinations of RPA primers (4 different primer pairs; 6 pairwise combinations). A multiplexed RPA is individually assessed with a LwaCas13a-based detection reaction, each programmed with a specific crRNA for the SARS-CoV-2 gene, and lateral-flow readout. Successful amplification was marked by production of a strong-colored band at a test band (T), which indicates target-activated Cas13a activity and resulting in efficient cleavage of the FAM-biotin reporter.

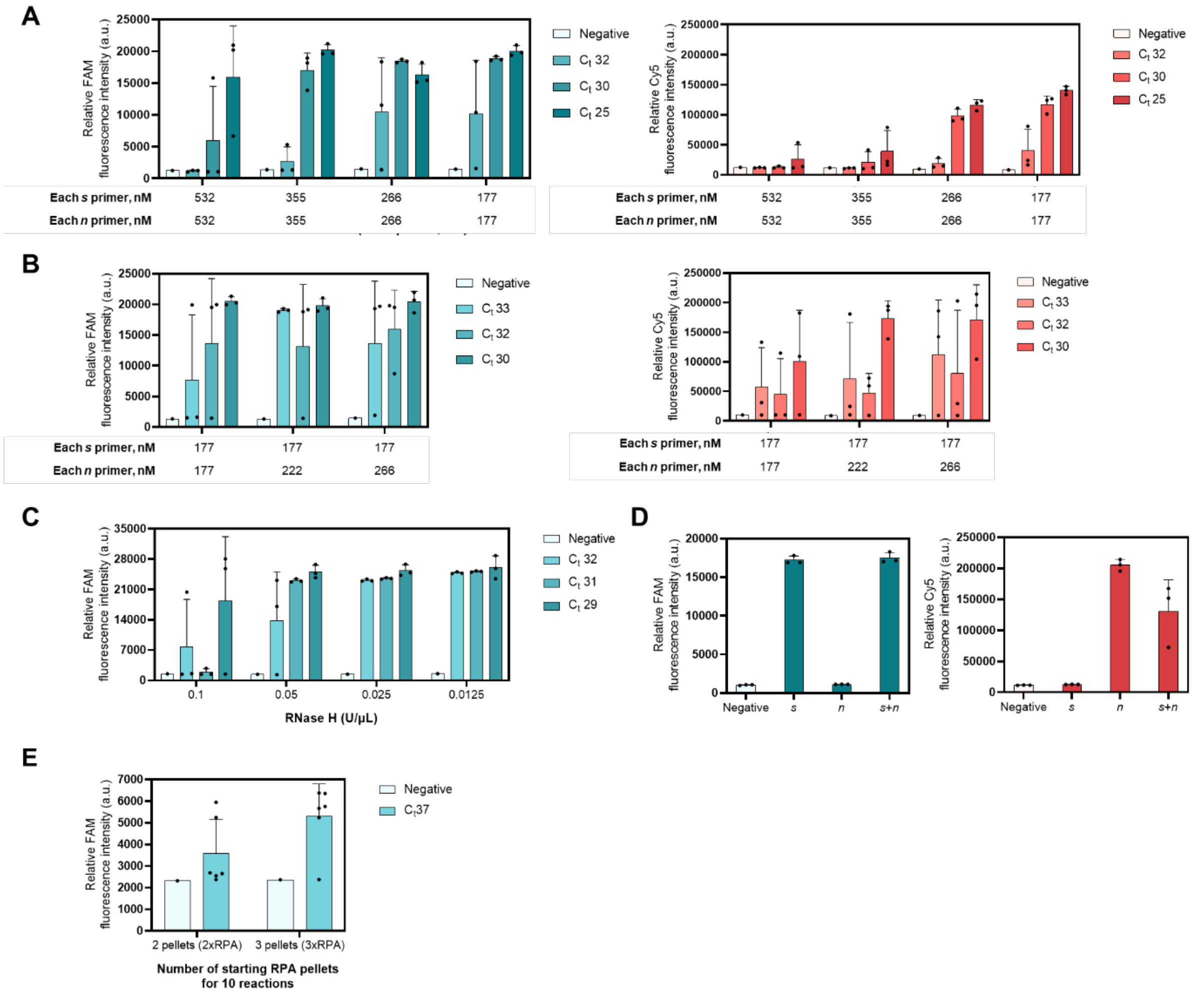

Figure S2: Evaluation of the sensitivity and specificity of the Multiplexed RT-RPA assay with real-time fluorescence detection.

(**A**) Optimizing total primer concentrations in multiplexed RPA with 1:1 malar ratio of *n* to *s* primer. Multiplexed RPA for the *s* and *n* gene of SARS-CoV-2 was performed using indicated primer concentrations and serially diluted SARS-CoV-2 genomic RNA template. In the second step, generated *s* and *n* amplicons were detected using a multiplexed CRISPR-Cas reaction containing LwaCas13a and PsmCas13b enzymes. LwaCas13a is programmed with a crRNA targeting the *s* amplicon and cleaves a FAM/IABkFQ -functionalized polyU reporter once target-activated (left), while PsmCas13b is programmed with a crRNA targeting the *n* amplicon and cleaves a Cy5/IABkRQ-functionalized polyA reporter (right). (**B**) Fine-tuning primer concentrations in the multiplexed RPA reaction. Multiplexed RPA for the *s* and *n* gene of SARS-CoV-2 was performed using indicated primer concentrations, with serially diluted SARS-CoV-2 genomic RNA as a template. Amplicons were detected in a multiplexed CRISPR-Cas reaction, via FAM fluorescence generated from *s*-targeted LwaCas13a (left) and Cy5 fluorescence from N-targeted PsmCas13b (right). (**C**) Optimizing RNase H concentrations in the RT-RPA reaction. RT-RPA for *s* gene amplification from serially diluted SARS-CoV-2 RNA, followed by LwaCas13a-based detection, was used. (**D**) Orthogonality of LwaCas13a and PsmCas13b in a multiplexed CRISPR-Cas detection. RPA reactions were performed with only *s* primers (*s*), only

n primers (*n*), or combined *s* and *n* primers (*s*+*n*). Endpoint fluorescence intensities of the multiplexed detection reactions (90 min) containing all components for LwaCas13-based detection of the *s* gene and PsmCas13b-based detection of the *n* gene were shown. Generated FAM (left) and Cy5 (right) fluorescence indicated cleavage of the reporters by the *s*-targeted LwaCas13a and the *n*-targeted PsmCas13b, respectively. For C-F, negative controls have no SARS-CoV-2 RNA template input. Data are mean ± s.d. from 3 replicates. (**E**) Analytical sensitivity of increasing of RPA pellet. Multiplexed RT-RPA for the *s* and *n* gene of SARS-CoV-2 was performed using indicated number of RPA pellets, with diluted SARS-CoV-2 genomic RNA at C_t 37 as a template. Subsequently, the amplicons were detected via FAM fluorescence generated from LwaCas13a-based reaction programmed with *s*- and *n*-targeted crRNAs.

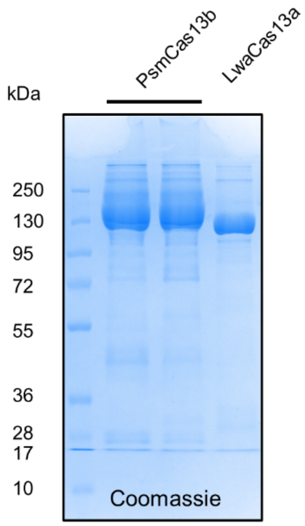

Figure S3: Protein purification of LwaCas13a and PsmCas13b.
12% SDS-PAGE gel of purified PsmCas13b and LwaCas13a. Each lane was loaded with 6 µg of protein. PsmCas13b was loaded twice.

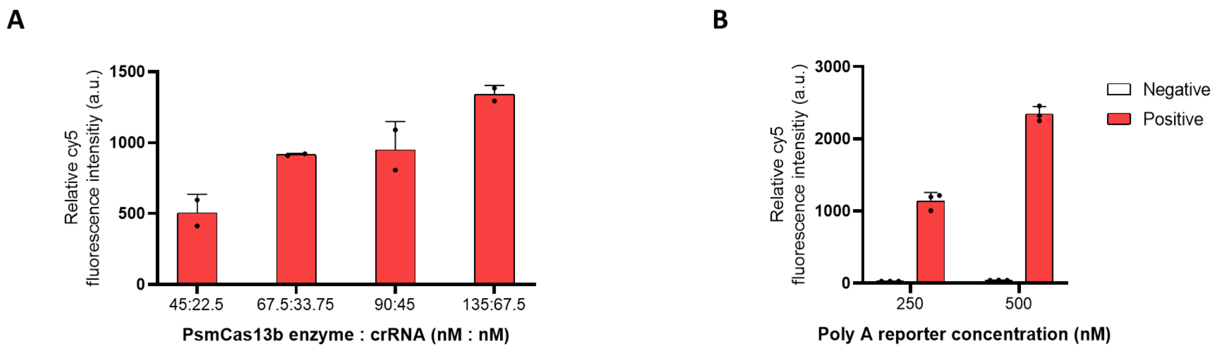

Figure S4: Optimizing PsmCas13b-based detection of the SARS-CoV-2 N gene.
(**A**) Varying PsmCas13b enzyme and crRNA amount in the detection reaction. The concentration of Cy5-functionalized polyA reporter was 250 nM in all conditions in (A). (**B**) Varying the amount of Cy5-polyA reporter in the detection reaction. 135 nM PsmCas13b and 67.5 nM crRNA were used for all conditions in (B). The *n* amplicon from the same RPA reaction was used as a substrate for all PsmCas13b-based detection reactions, which were performed for 90 min at 37 ºC, without any special additive in the RPA nor the CRISPR-Cas reactions. Endpoint fluorescence intensities were shown. Negative control reactions in (B) have no SARS-CoV-2 RNA template input. Error bars, ± s.d. from 2 replicates (A) and 3 replicates (B).

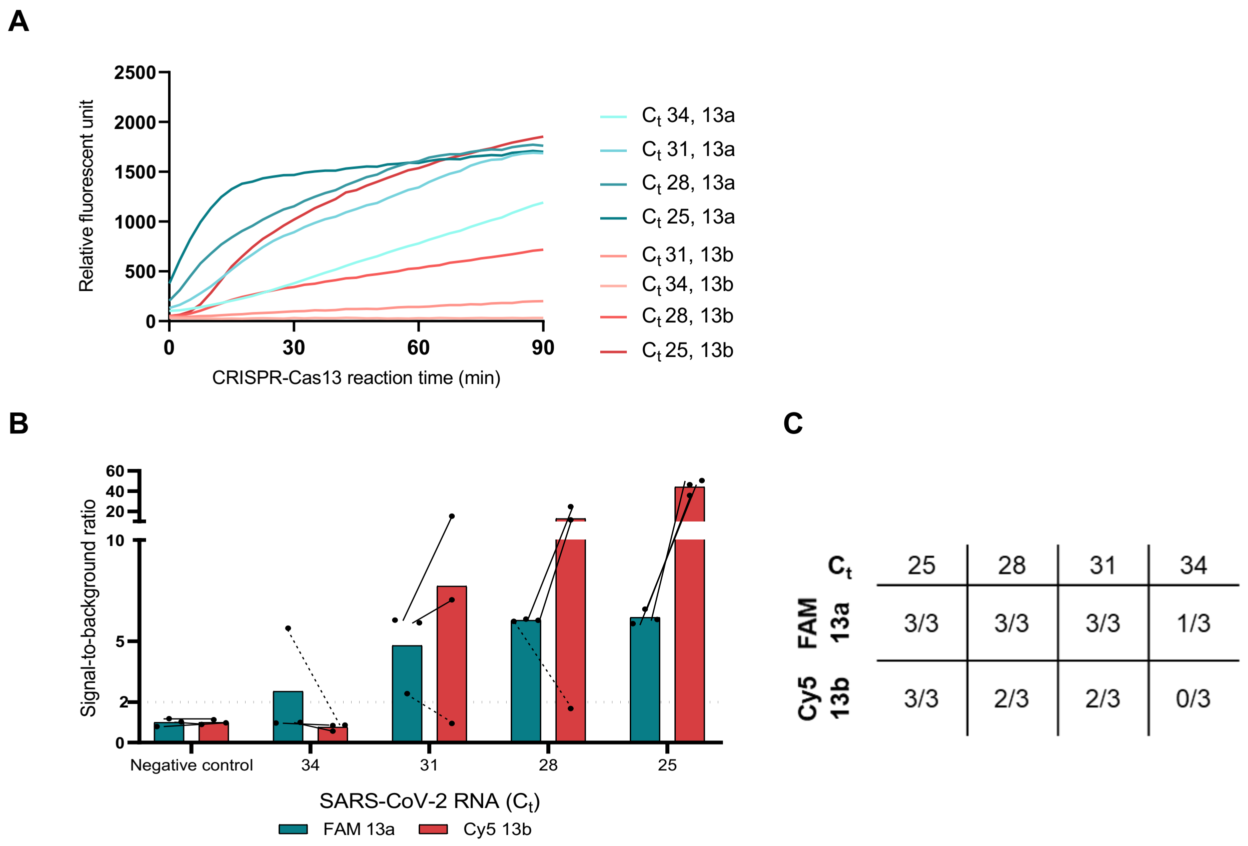

Figure S5: Comparison of LwaCas13a- and PsmCas13b-based detection.
The *n* amplicons from the RPA reactions performed with serially diluted SARS-CoV-2 RNA were used as substrates for all Cas13-based detection reactions, which were performed for 90 min at 37 ºC. The concentrations of all other components in the CRISPR-Cas reactions were identical, and no special additive was added to the RPA nor the CRISPR-Cas reactions. (**A**) Kinetics of fluorescence signal generation from PsmCas13b-mediated detection is slower than that of LwaCas13a. (**B**) When we calculated signal-to-noise (S/N, with noise defined as fluorescence intensity obtained from a no input sample performed in parallel), PsmCas13b generates higher signal-to-noise than LwaCas13a, primarily due to lower background fluorescence in the negative control sample for PsmCas13b. However, LwaCas13a-mediated detection still has higher positive rates **(B, C)** than PsmCas13b. Error bars, ± s.d. from 3 replicates.

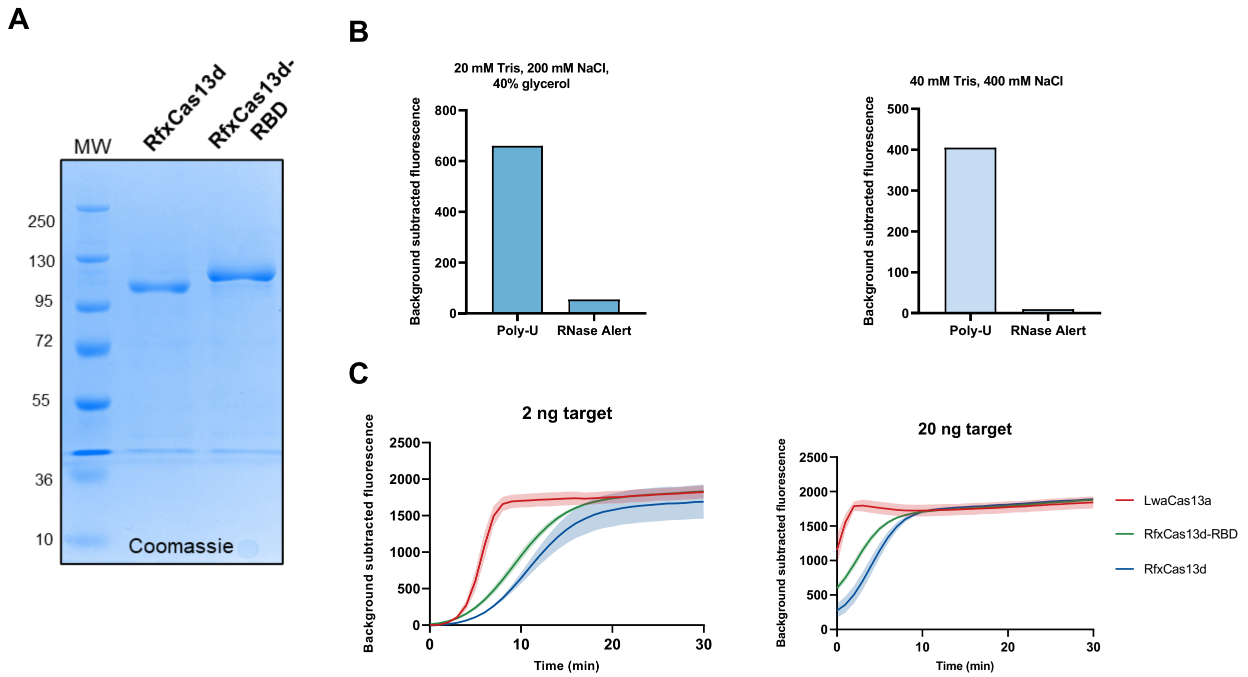

Figure S6: RfxCas13d-based detection of SARS-CoV-2 RNA.
(**A**) SDS-PAGE gel of purified RfxCas13d (112 kDa) and RfxCas13d-RBD (120 kDa). Each lane was loaded with 2 ng of protein. (**B**) RfxCas13d exhibited collateral cleavage preference in degrading a polyU reporter over RNaseAlert®. An *n* gene amplicon of SARS-CoV-2 was used as a substrate in a Cas13d-mediated detection. Two reaction conditions as well as two potential collateral reporters of RfxCas13d were tested. Fluorescence signal generated after 60 min of the CRISPR-Cas reaction for each condition (performed once) is shown. (**C**) Comparison of LwaCas13a, RfxCas13d, and RfxCas13d-RBD in SHERLOCK detection of the SARS-CoV-2 *n* gene. RfxCas13d-RBD has improved collateral cleavage efficiency over RfxCas13d, but still does not match LwaCas13a. Fluorescence signal generated over time using the FAM-polyU reporter is shown, at 2 ng (left) and 20 ng (right) RNA input amount. Error bands in (C) ± s.d. from 3 replicates.

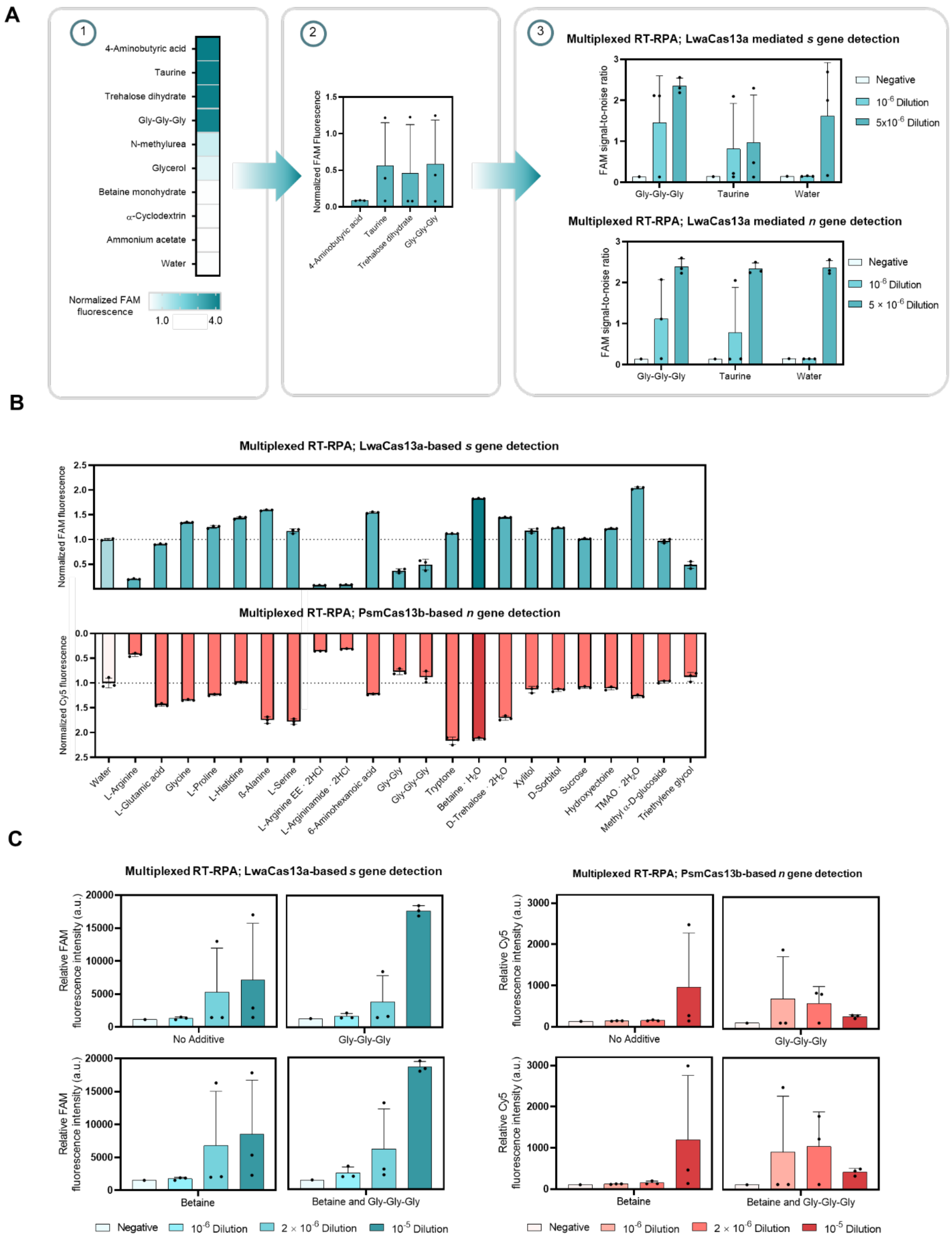

Figure S7: Screening of additives to enhance multiplexed RPA and CRISPR-Cas reactions.

(**A**) Screening for additives that enhance multiplexed RT-RPA. Left: nine additives were assessed in a conventional two-step (RPA, then LwaCas13a-based CRISPR-Cas) SHERLOCK detection for the *s* gene of SARS-CoV-2. Middle: four active additives were evaluated further in triplicates, using less RNA input. Right: two best-performing additives—triglycine and taurine—were finally assessed in a multiplexed RPA reaction for the *s* and *n* gene of SARS-CoV-2. Two serial dilutions of SARS-CoV-2 RNA were used as a template; negative control has no SARS-CoV-2 RNA template input. Generated *s* and *n* amplicons were detected in separate LwaCas13a-based detection reactions, whose FAM fluorescence signal is shown. (**B**) Screening for additives that improve the multiplexed CRISPR reaction. Twenty-two additives were assessed in the multiplexed CRISPR-Cas detection, using an RPA product from a multiplexed. Endpoint FAM (Qian et al.) and Cy5 (bottom) fluorescence intensities were normalized against intensities obtained from the no-additive controls. Data are mean ± s.d. from 3 replicates. L-Arginine EE. 2HCl, L-Arginine ethyl ester dihydrochloride. TMAO, trimethylamine N-oxide. (**C**) Cumulative benefits of triglycine additive in the multiplexed RPA and betaine monohydrate additive in the multiplexed CRISPR-Cas reaction. Multiplexed RPA to amplify the *s* and *n* genes of SARS-CoV-2 was performed in the presence or absence of 40 mM triglycine, using serially diluted SARS-CoV-2 RNA as a template, and multiplexed RPA was allowed to proceed for 25 min at 42 ºC. Multiplexed CRISPR-Cas reactions to detect the *s* and *n* amplicons were then performed in the presence or absence of 500 mM betaine. FAM and Cy5 fluorescence signal (indicative of Cas13a-mediated *s* gene and Cas13b-mediated *n* gene detection respectively) is shown. RNase-free water was used as input of all negative control reactions. Data are mean ± s.d. from 3 replicates.

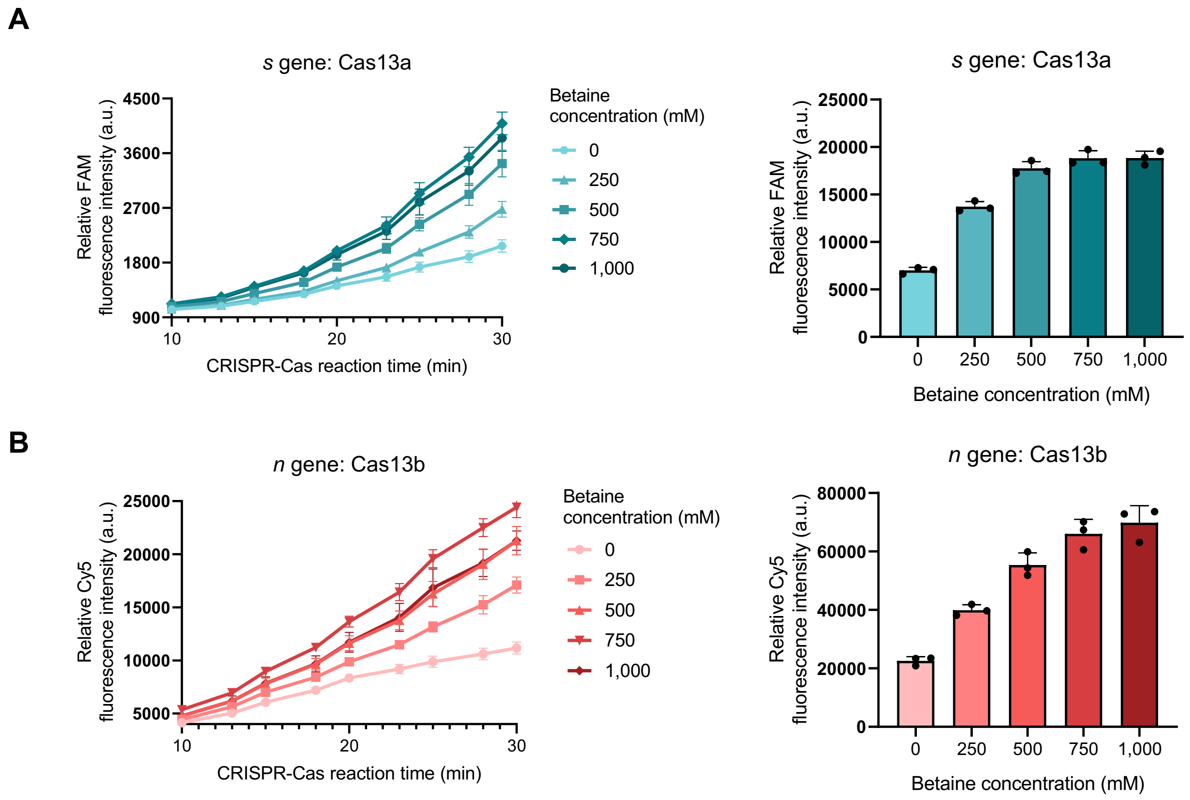

Figure S8: Optimizing betaine monohydrate concentration in multiplexed CRISPR-Cas detection.

FAM fluorescence indicated cleavage of the FAM reporter by the *s*-targeted LwaCas13a, while Cy5 fluorescence was from cleavage of the Cy5 reporter by the *n*-targeted PsmCas13b. FAM (**A**) and Cy5 (**B**) fluorescence signal generated after 30 min of the multiplexed CRISPR-Cas reaction for each condition are shown. The target was a diluted RPA product at 1:300 dilution. Error bars, ± s.d. from 3 replicates.

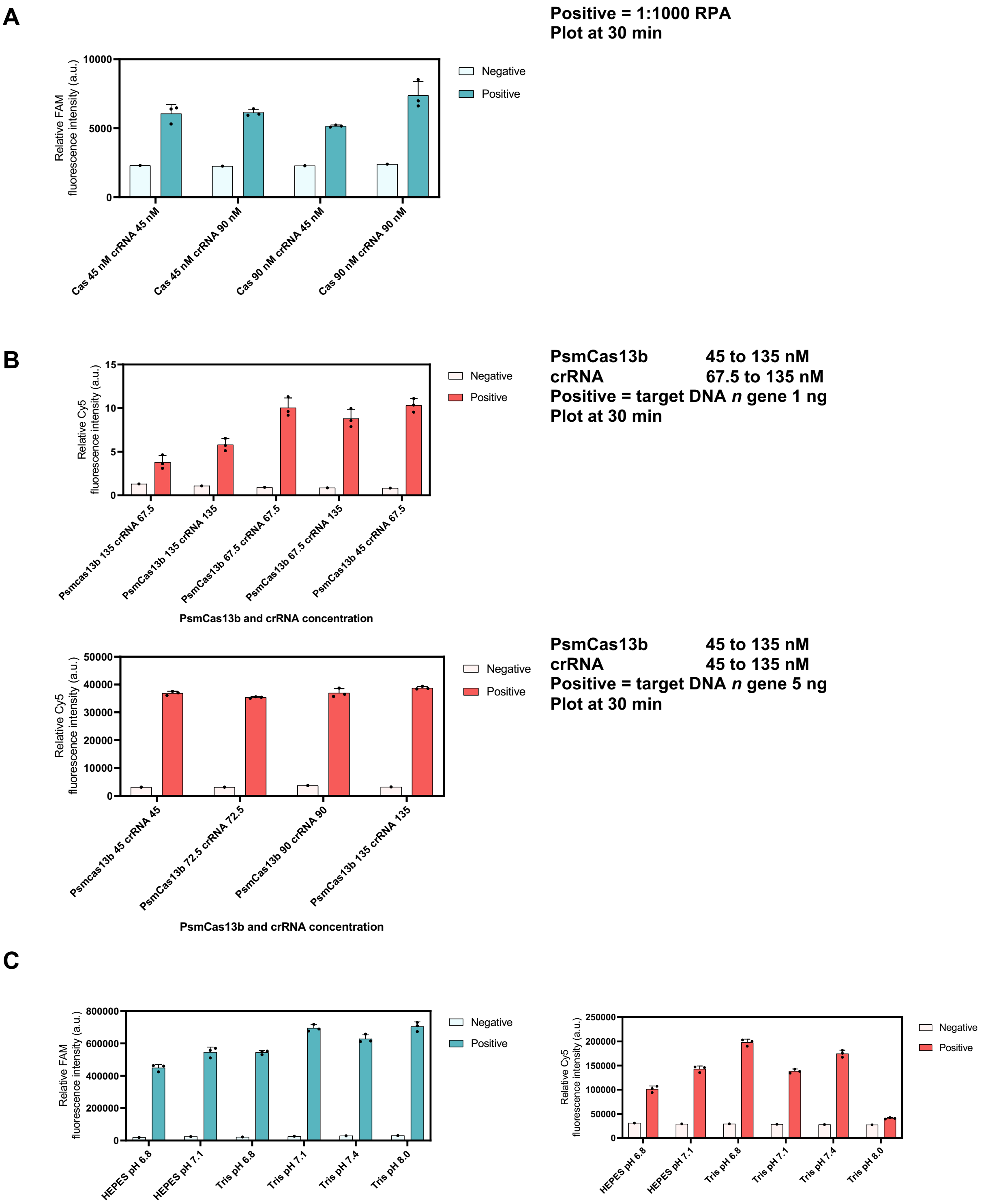

Figure S9: Effect of crRNA ratio and pH in multiplexed CRISPR-Cas 13 based detection
(**A**) LwaCas13a-based detection of a diluted RPA product at different concentration of LwaCas13a (45 – 90 nM) and LwaCas13a-crRNA (45 – 90 nM) for the *s* gene.

(**B**) PsmCas13b-based detection of purified N amplicon at different concentration of PsmCas13b (45 – 135 nM) and PsmCas13b-crRNA (45 – 135 nM) for the *n* gene.

(**C**) Identifying optimal buffer conditions for LwaCas13a and PsmCas13b. The multiplexed CRISPR-based detection reaction prepared in Tris-HCl or HEPES buffer (pH 6.8 – 8.0) were performed using a diluted multiplexed RPA product as input. Error bars, ± s.d. from 3 replicates.

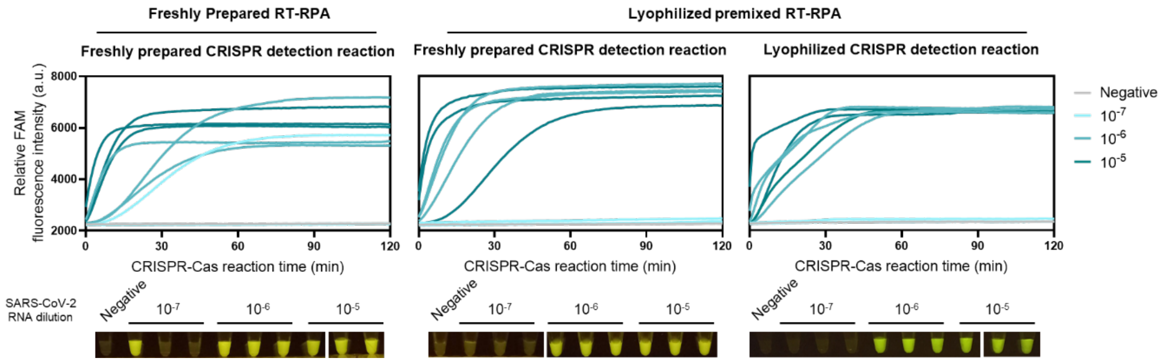

Figure S10: lyophilized RT-RPA for multiplexed amplification of the S and N genes

Left vs middle graphs: all-in-one lyophilized RT-RPA for multiplexed amplification of the *s* and *n* genes has similar sensitivity as freshly prepared RT-RPA. Non-volatile salt KOAc (the high concentration of which likely destabilizes protein components of RT-RPA upon lyophilization) and the typical reaction initiator $Mg(OAc)_2$ were excluded, while all other components required for multiplexed RT-RPA including all protein components for RPA, reverse transcriptase, RNase H, primer pairs, and other substrates and buffering/crowding compounds can be lyophilized together. The lyophilized RT-RPA pellets were reconstituted with 12.7 µl of serially diluted SARS-CoV-2 RNA, $Mg(OAc)_2$, and KOAc, and incubated at 42°C for 25 min. Thereafter, the multiplexed RPA products were used in a freshly prepared Cas13a-based detection with *s*- and *n*-targeted crRNAs. FAM fluorescence generated over 120 min for each condition is shown. Middle vs right graphs: lyophilized LwaCas13a-based detection reaction has similar sensitivity as freshly prepared reactions.

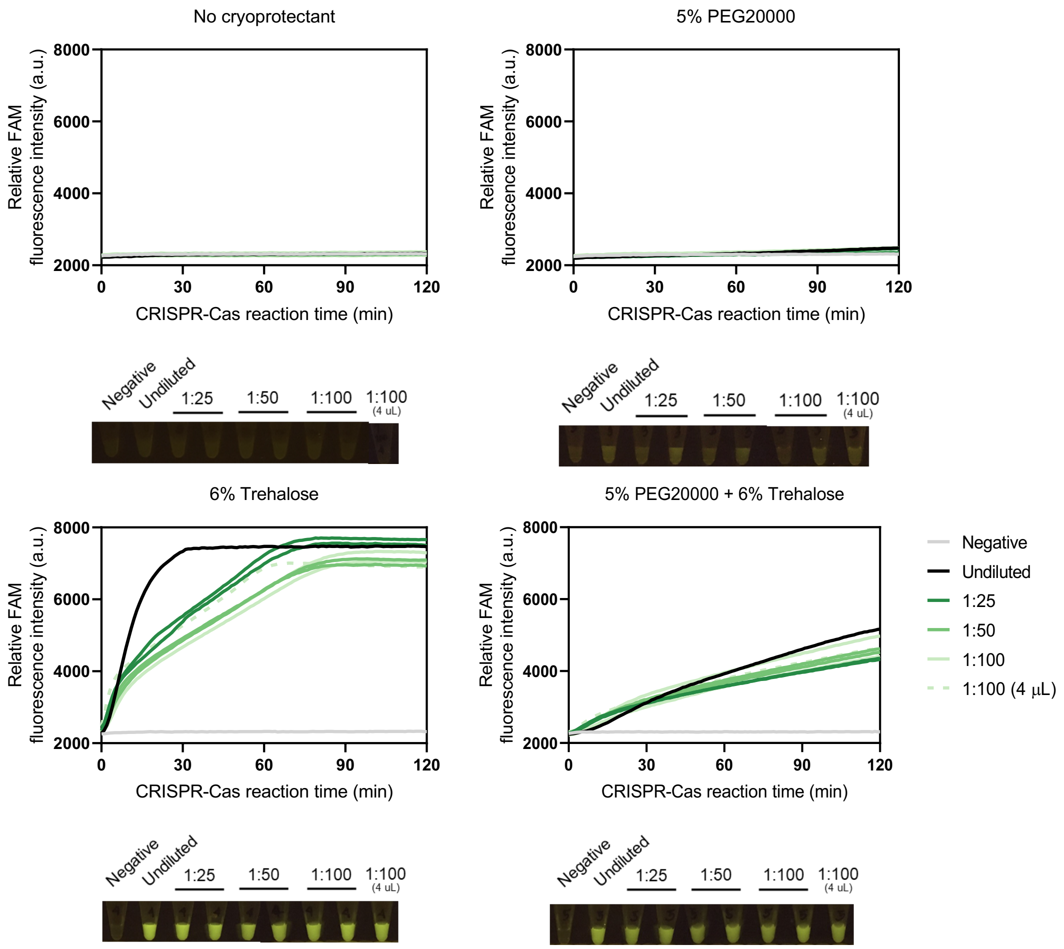

Figure S11: Effects of cryoprotectants on lyophilized CRISPR-Cas13a reactions. LwaCas13a-based detection reactions were prepared with or without 5% (w/v) PEG20000 and 6% (w/v) trehalose added as a cryoprotectant. $MgCl_2$ was omitted from all reactions and was added at the rehydration step. The detection was performed at 37 °C using *n* gene RPA product at various dilution levels and monitored under FAM channel using a real-time thermal cycler. The input volume of the RPA dilutions was 2 µL except for the 1:100 dilution sample in which we used either 2-µL or 4-µL input volume. The end-point fluorescence in tubes was visualized using a BluPAD transilluminator.

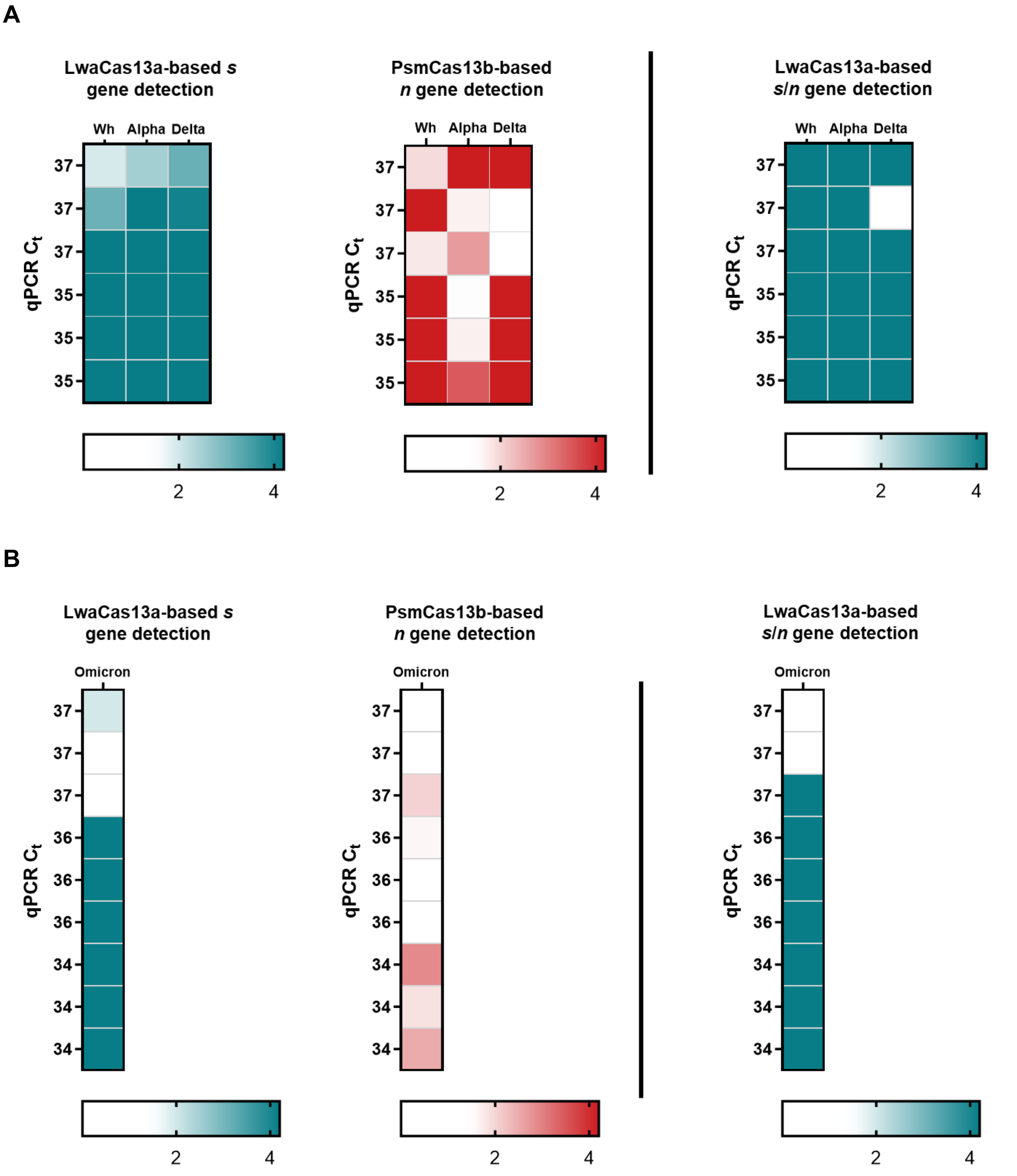

Figure S12: The lyophilized multiplexed CRISPR-based detection is robust across major SARS-CoV-2 variants. Performance of the multiplexed CRISPR-based detection for **(A)** ancestral Wuhan strain, Alpha variant, Delta variant, and **(B)** Omicron variant. FAM and Cy5

fluorescence intensities at 60 minutes were normalized against averaged intensities obtained from the no template control.

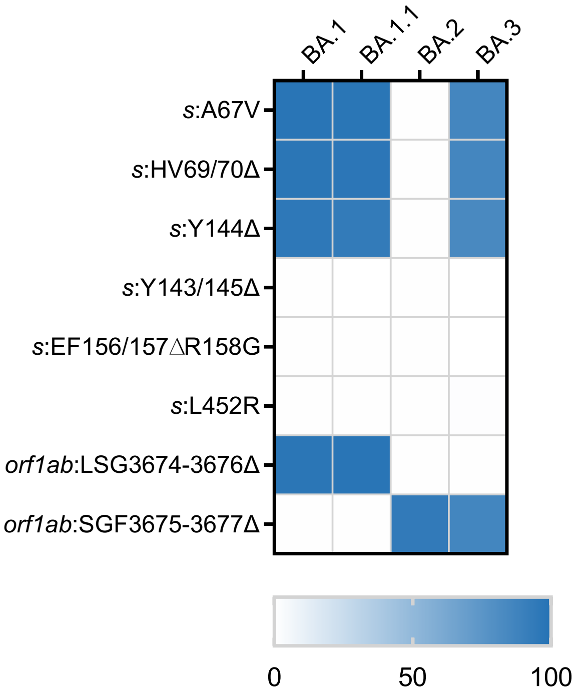

Figure S13: Mutation prevalence across Omicron sub-lineages
Percentages of mutations prevalence across four SARS-CoV-2 Omicron sub-lineages (BA.1, BA.1.1, BA.2, and BA.3) were obtained from Outbreak.info as of 15 March 2022; BA.1 (n = 1,005,355), BA.1.1 (n = 827,907), BA.2 (n = 316,708), and BA.3 (n = 694))

		crRNA		%F
**SGF3675-3677Δ**	3′	**tatgatcaaac---------ttcgattttctgacaca**	5′	-
Wuhan	5′	atactagtttgtctggttttaagctaaaagactgtgt	3′	-
Alpha	5′	atactagtttg---------aagctaaaagactgtgt	3′	94.0
Beta	5′	atactagtttg---------aagctaaaagactgtgt	3′	96.2
Gamma	5′	atactagtttg---------aagctaaaagactgtgt	3′	93.1
Delta	5′	atactagtttgtctggttttaagctaaaagactgtgt	3′	99.0
Omicron	5′	atactagtttt---------aagctaaaagactgtgt	3′	85.4
**HV69/70Δ**	3′	**aaccaaggtacgat------agagaccctggtta**	5′	-
Wuhan	5′	ttggttccatgctatacatgtctctgggaccaat	3′	-
Alpha	5′	ttggttccatgcta------tctctgggaccaat	3′	88.1
Beta	5′	ttggttccatgctatacatgtctctgggaccaat	3′	92.3
Gamma	5′	ttggttccatgctatacatgtctctgggaccaat	3′	96.6
Delta	5′	ttggttccatgctatacatgtctctgggaccaat	3′	96.9
Omicron	5′	ttggttccatgtta------tctctgggaccaat	3′	84.5*
*%F for sublineage; BA.1 (95) and BA.2 (0)				
**Y144Δ**	3′	**aaaacccacaaa---tggtgtttttgttgtt**	5′	-
Wuhan	5′	ttttgggtgtttattaccacaaaaacaacaa	3′	-
Alpha	5′	ttttgggtgttt---accacaaaaacaacaa	3′	86.1
Beta	5′	ttttgggtgtttattaccacaaaaacaacaa	3′	100
Gamma	5′	ttttgggtgtttattaccacaaaaacaacaa	3′	100
Delta	5′	ttttggatgtttattaccacaaaaacaacaa	3′	62.8
Omicron	5′	ttttgg---------accacaaaaacaacaa	3′	85.0
**EF156/157ΔR158G**	3′	**ttcaacctacctttcac------ctcaaataaga**	5′	-
Wuhan	5′	aagttggatggaaagtgagttcagagtttattct	3′	-
Alpha	5′	aagttggatggaaagtgagttcagagtttattct	3′	99.0
Beta	5′	aagttggatggaaagtgagttcagagtttattct	3′	100
Gamma	5′	aagttggatggaaagtgagttcagagtttattct	3′	100
Delta	5′	aagttggatggaaagtg------gagtttattct	3′	98.8
Omicron	5′	aagttggatggaaagtgagttcagagtttattct	3′	100
**L452R**	3′	**ttccaaccaccattaatattaatagcca**	5′	-
Wuhan	5′	aaggttggtggtaattataattacctgt	3′	-
Alpha	5′	aaggttggtggtaattataattacctgt	3′	98.0
Beta	5′	aaggttggtggtaattataattacctgt	3′	100
Gamma	5′	aaggttggtggtaattataattacctgt	3′	96.6
Delta	5′	aaggttggtggtaattataattacggt	3′	96.8
Omicron	5′	aaggttagtggtaattataattacctgt	3′	84.7

Figure S14: Multiple sequence alignments of the guide RNA spacer targeting a specific mutation representative of SARS-CoV-2 variant sequences. %F, frequency of the sequence among the variant genomes. The associated dataset was given in Figure 3–source data 2. *Black shading highlights the mismatches to the corresponding crRNA. The underlines highlight the anticipated targeted variants and targeted sequences of each design.*

	Forward Primer	%F	Reverse Primer	%F
**SGF3675-3677Δ**	5′ **agttgggtgatgcgtattatgacatggttg** 3′	-	3′ **catacgtagtcgacatcacaatgattagga** 5′	-
Wuhan	5′ agttgggtgatgcgtattatgacatggttg 3′	-	5′ gtatgcatcagctgtagtgttactaatcct 3′	-
Alpha	5′ agttgggtgatgcgtattatgacatggttg 3′	100	5′ gtatgcatcagctgtagtgttactaatcct 3′	99.0
Beta	5′ agttgggtgatgcgtattatgacatggttg 3′	100	5′ gtatgcatcagctgtagtgttactaatcct 3′	100
Gamma	5′ agttgggtgatgcgtattatgacatggttg 3′	100	5′ gtatgcatcagctgtagtgttactaatcct 3′	100
Delta	5′ agttgggtgatgcgtattatgacatggttg 3′	100	5′ gtatgcatcagctgt gtgttactaatcct 3′	90.0
Omicron	5′ agttgggtgatgcgtattatgacatggttg 3′	100	5′ gtatgcatcagctgtagtgttactaatcct 3′	99.8
**HV69/70Δ**	5′ **attaccctgacaaagttttcagatcctcag** 3′	-	3′ **ggacaggatggtaaattactaccacaaata** 5′	-
Wuhan	5′ attaccctgacaaagttttcagatcctcag 3′	-	5′ cctgtcctaccatttaatgatggtgtttat 3′	-
Alpha	5′ attaccctgacaaagttttcagatcctcag 3′	100	5′ cctgtcctaccatttaatgatggtgtttat 3′	99.0
Beta	5′ attaccctgacaaagttttcagatcctcag 3′	100	5′ cctgtcctaccatttaatgatggtgtttat 3′	100
Gamma	5′ attaccctgacaaagttttcagatcctcag 3′	100	5′ cctgtcctaccatttaatgatggtgtttat 3′	100
Delta	5′ attaccctgacaaagttttcagatcctcag 3′	99.8	5′ cctgtcctaccatttaatgatggtgtttat 3′	99.0
Omicron	5′ attaccctgacaaagttttcagatcctcag 3′	99.8	5′ cctgtcctaccatttaatgatggtgtttat 3′	99.8
**Y144Δ**	5′ **acgctactaatgttgttattaaagtctgtg** 3′	-	3′ **tcaaataagatcacgcttattaacgtgaaa** 5′	-
Wuhan	5′ acgctactaatgttgttattaaagtctgtg 3′	-	5′ agtttattctagtgcgaataattgcacttt 3′	-
Alpha	5′ acgctactaatgttgttattaaagtctgtg 3′	99.0	5′ agtttattctagtgcgaataattgcacttt 3′	100
Beta	5′ acgctactaatgttgttattaaagtctgtg 3′	100	5′ agtttattctagtgcgaataattgcacttt 3′	100
Gamma	5′ acgctactaatgttgttattaaagtctgtg 3′	100	5′ agtttattctagtgcgaataattgcacttt 3′	100
Delta	5′ acgctactaatgttgttattaaagtctgtg 3′	99.4	5′ agtttattctagtgcgaataattgcacttt 3′	99.6
Omicron	5′ acgctactaatgttgttattaaagtctgtg 3′	100	5′ agtttattctagtgcgaataattgcacttt 3′	99.8
**EF156/157ΔR158G**	5′ **Same as Y144Δ** 3′	-	3′ **attaacgtgaaaacttatacagagagtcgg** 5′	-
Wuhan	5′ Same as Y144Δ 3′	x	5′ taattgcacttttgaatatgtctctcagcc 3′	-
Alpha	5′ Same as Y144Δ 3′	x	5′ taattgcacttttgaatatgtctctcagcc 3′	100
Beta	5′ Same as Y144Δ 3′	x	5′ taattgcacttttgaatatgtctctcagcc 3′	100
Gamma	5′ Same as Y144Δ 3′	x	5′ taattgcacttttgaatatgtctctcagcc 3′	100
Delta	5′ Same as Y144Δ 3′	x	5′ taattgcacttttgaatatgtctctcagcc 3′	99.1
Omicron	5′ Same as Y144Δ 3′	x	5′ taattgcacttttgaatatgtctctcagcc 3′	100
**L452R**	5′ **acaggctgcgttatagcttggaattctaac** 3′	-	3′ **ccatatctaacaaatccttcagattagagt** 5′	-
Wuhan	5′ acaggctgcgttatagcttggaattctaac 3′	-	5′ tgtatagattgtttaggaagtctaatctca 3′	-
Alpha	5′ acaggctgcgttatagcttggaattctaac 3′	100	5′ tgtatagattgtttaggaagtctaatctca 3′	100
Beta	5′ acaggctgcgttatagcttggaattctaac 3′	96.2	5′ tgtatagattgtttaggaagtctaatctca 3′	100
Gamma	5′ acaggctgcgttatagcttggaattctaac 3′	100	5′ tgtatagattgtttaggaagtctaatctca 3′	100
Delta	5′ acaggctgcgttatagcttggaattctaac 3′	99.8	5′ ggtatagattgtttaggaagtctaatctca 3′	98.8
Omicron	5′ acaggctgcgttatagcttggaattctaac 3′	99.5	5′ tgtatagattgtttaggaagtctaatctca 3′	99.8

Figure S15: Multiple sequence alignments of primer pairs targeting a specific mutation representative of SARS-CoV-2 variant sequences. %F, frequency of the sequence among the variant isolates. The associated dataset was given in Figure 3–source data 2. *Black shading highlights the mismatches to the corresponding crRNA. The underlines highlight the anticipated targeted variants and targeted sequences of each design.*

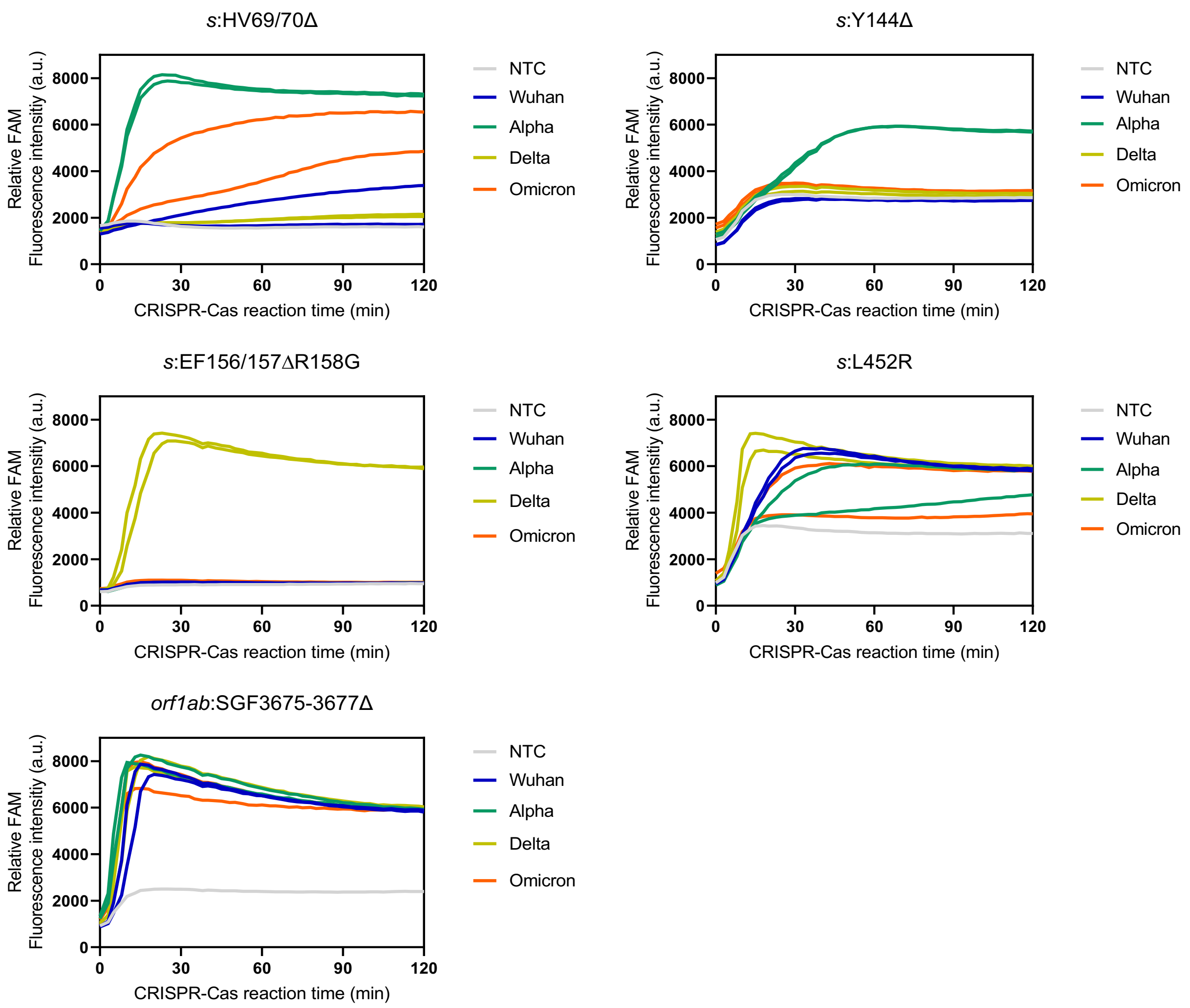

Figure S16: **Kinetic tracing of CRISPR-Cas13a detection reaction underlying Figure 3B** Screening of primer sets and crRNA for cross-reactivity against the high viral RNA titers (C_t 28) of ancestral Wuhan, the alpha, and the delta strains, as well as the omicron strain. Kinetics of FAM fluorescence signal generation from the SARS-CoV-2 gene detection (as indicated) via a singleplexed CRISPR-Cas13a reaction, using cultured SARS-CoV-2 viral RNA extracts, except the omicron RNA extract from clinical sample, verified to be positive with different coronaviruses via RT-qPCR. RNase-free water was used as input of all negative control reactions.

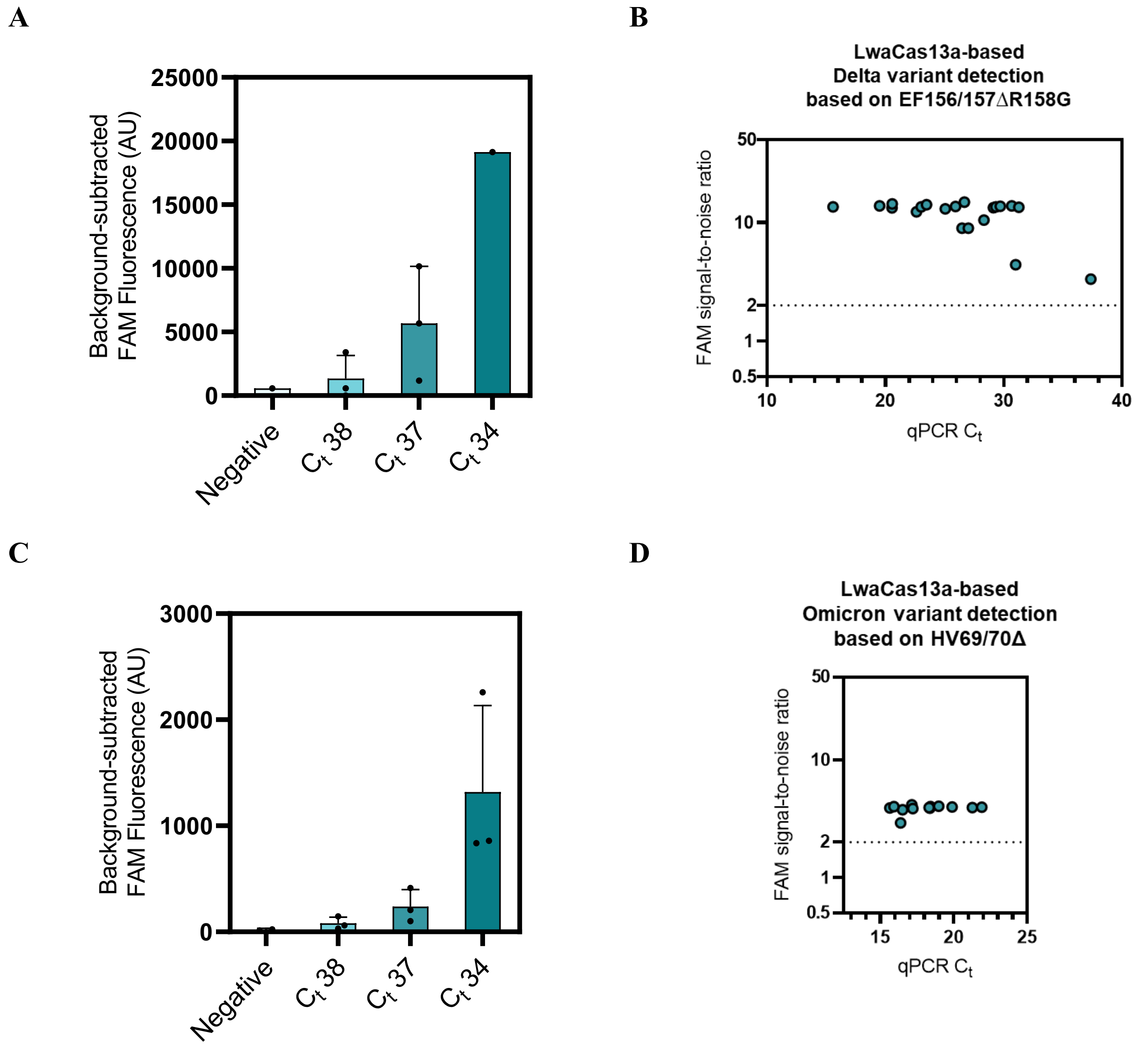

Figure S17: Singleplexed Detection of SARS-CoV-2 Delta and Omicron variants.
(A) LwaCas13a-based Delta variant detection based on EF156/157ΔR158G performed on a dilution series of a SARS-CoV-2 Delta RNA extract with determined C_t value. FAM fluorescence at 60 minutes were subtracted with background signal generated in negative control whose input is RNase-free water. Data are mean ± s.d. from 3 replicates. **(B)** LwaCas13a-based Delta variant detection based on EF156/157ΔR158G performed on Clinical Delta samples with determined C_t value. FAM fluorescence at 60 minutes were normalized against intensities obtained from the no template control. **(C)** LwaCas13a-based Omicron variant detection based on HV69/70Δ performed on a dilution series of a SARS-CoV-2 Omicron RNA extract with determined C_t value. FAM fluorescence at 60 minutes were subtracted with background signal generated in negative control whose input is RNase-free water. Data are mean ± s.d. from 3 replicates. **(D)** LwaCas13a-based Omicron variant detection based on HV69/70Δ performed on Clinical Delta samples with determined C_t value. FAM fluorescence at 60 minutes were normalized against intensities obtained from the no template control.

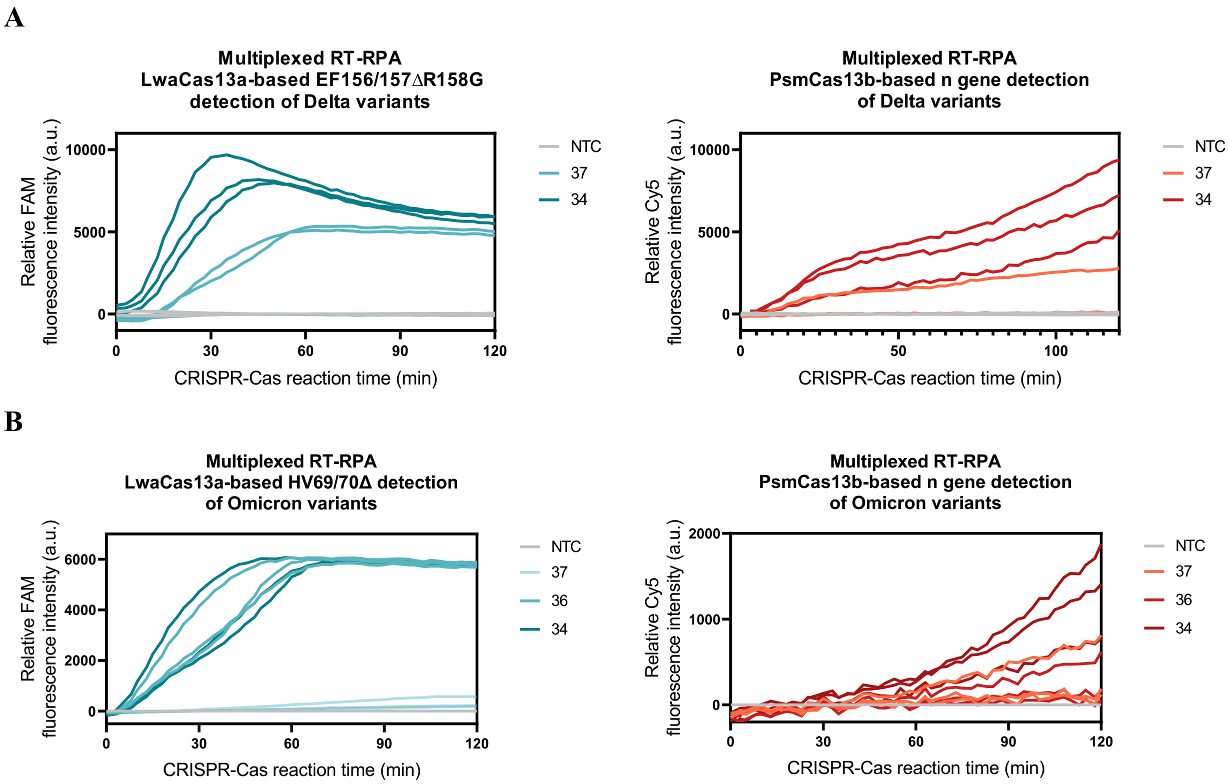

Figure S18: Kinetic traces of CRISPR-Cas13a/b detection reactions for Figure 3C and 3E. **(A)** Multiplexed LwaCas13a-mediated EF156/157ΔR158G (*s* gene) and PsmCas13b-mediated pan-SARS-CoV-2 (*n* gene) detection **(B)** Multiplexed LwaCas13a-mediated HV69/70Δ (*s* gene) and PsmCas13b-mediated pan-SARS-CoV-2 (*n* gene) detection. Kinetics of FAM (left) and Cy5 (right) fluorescence signal generation via a multiplexed CRISPR-Cas13a/b reaction performed on a dilution series with determined C_t value of Delta RNA extracts from laboratory culture **(A)** or Omicron RNA extracts from clinical samples **(B)** were shown. RNase-free water was used as input of all no template control (NTC) reactions.

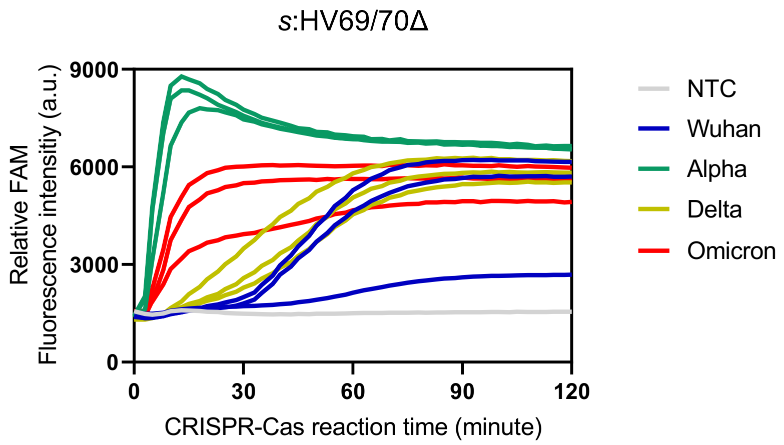

Figure S19: Exclusivity (cross-reactivity) testing of primer and crRNA targeting HV69/70Δ detection against the high viral RNA titers (C_t 25) of ancestral Wuhan, the alpha, and the delta strains, as well as the omicron strain
Kinetics of FAM fluorescence signal generation from the SARS-CoV-2 *s* gene harboring HV69/70Δ mutation detection via a singleplexed CRISPR-Cas13a reaction, using cultured SARS-CoV-2 viral RNA extracts, except the omicron RNA extract from clinical sample, verified to be positive with different coronaviruses via RT-qPCR. RNase-free water was used as input for negative control reactions.

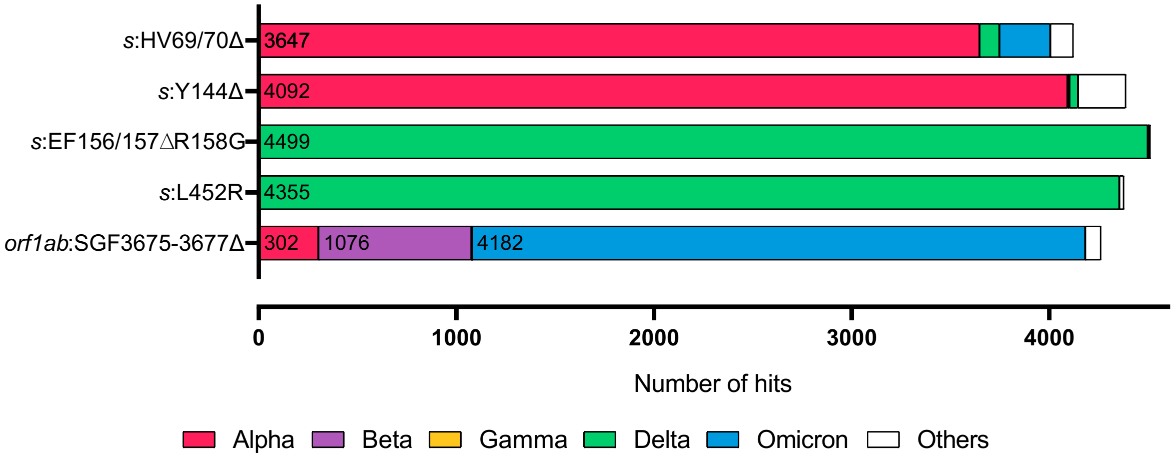

Figure S20: Exclusivity evaluation of designed crRNAs
Distribution of the SARS-COV-2 variant genome hits from BLAST search using crRNA spacer complementary sequences as queries. The associated dataset was given in Figure 3–source data 1.

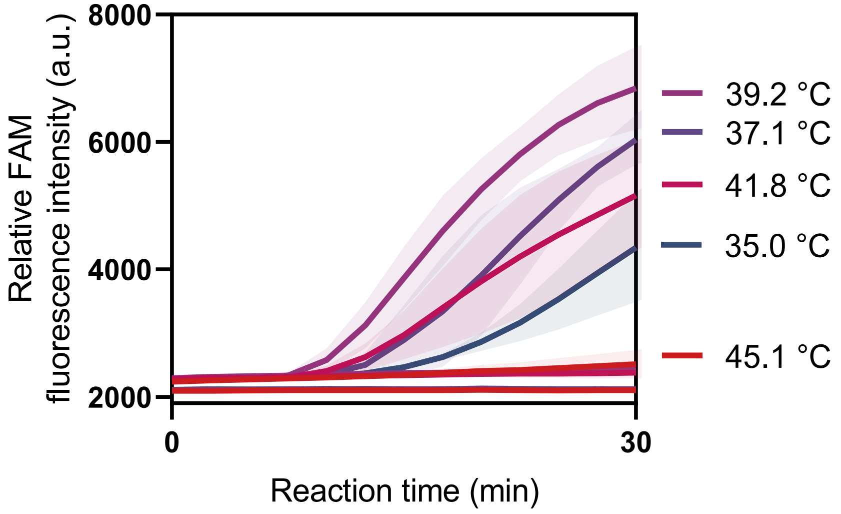

Figure S21: Effects of the reaction temperature on SHINE protocol.
A one-pot SHINE detection was performed using *s* gene primers and LwaCas13a-crRNA targeting *s* gene at varying temperature ranging from 35.0 - 45.1 °C. FAM fluorescence was monitored using a real-time PCR (CFX Connect Real-Time PCR System, Bio-Rad). Error bars, ± s.d. from 3 replicates.

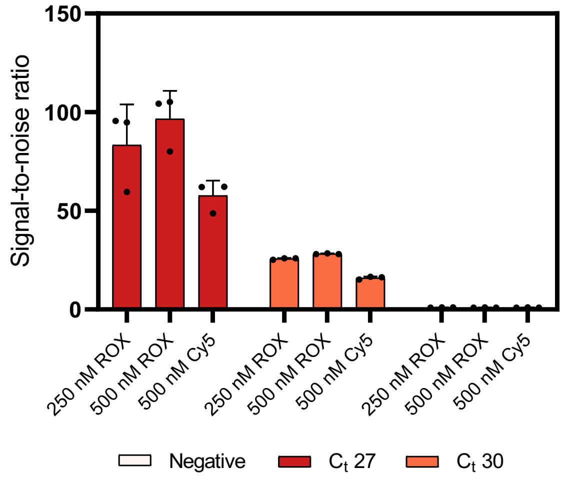

Figure S22: **Comparing ROX-based vs Cy5-based polyA reporter for PsmCas13b-based detection.**

PsmCas13b-based detection of RT-RPA products with serially diluted SARS-CoV-2 RNA as input was performed with either 250 nM Rhodamine X (ROX)-PolyA reporter, 500 nM ROX-PolyA, or 500 nM Cy5-PolyA reporter. Signal-to-noise ratios (defined as fluorescence intensities of the samples divided by those from negative input samples) generated after 22.5 min of the CRISPR-Cas reaction for each condition are shown. Error bars, ± s.d. from 3 replicates.

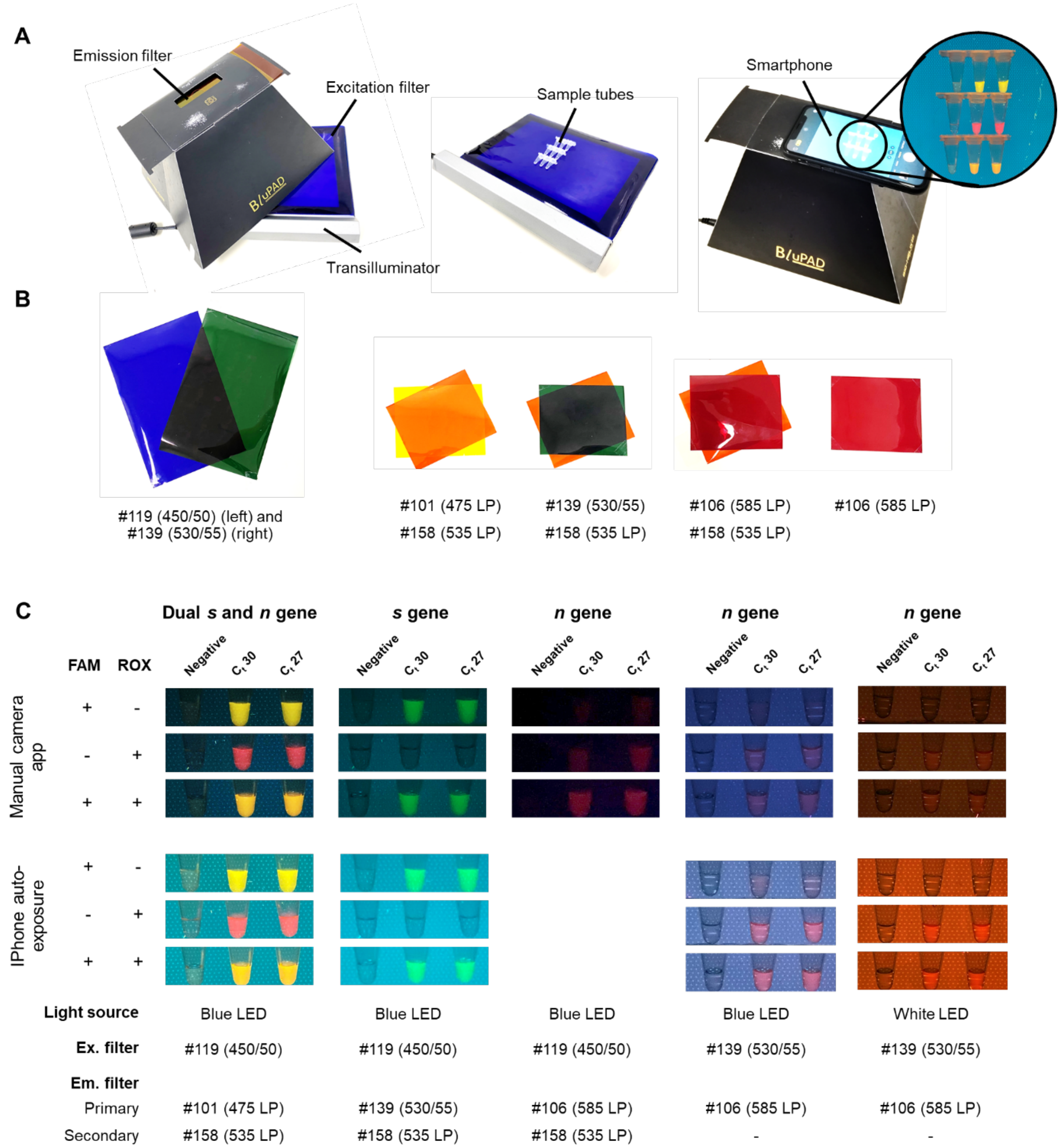

Figure S23: Direct-eye and smartphone-based visualization of multiplexed CRISPR-based detection.

(**A**) An example of equipment setup. We used a dual blue/white LED transilluminator. (**B**) Lighting gels with appropriate light filtering for visualization of FAM and ROX. (**C**) Images taken by manual camera application (Qian et al.) and auto-exposure (bottom) using different combinations of LED light source, excitation filters, emission filters, and phone-based image acquisition for visualization of FAM and ROX signals generated from multiplexed CRISPR-based detection reactions.

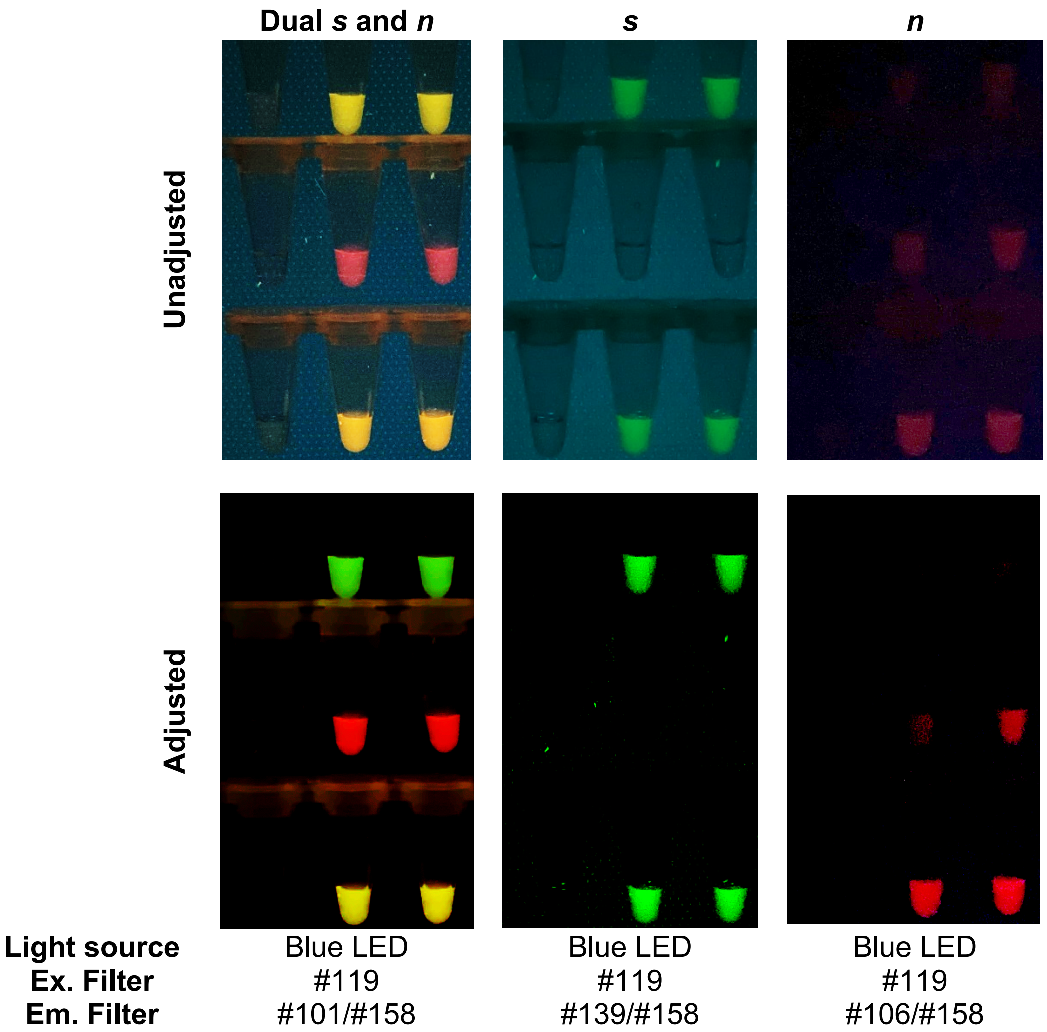

Figure S24: Raw smartphone images vs processed images of FAM/ROX-labeled multiplexed detection samples.

The acquired images were processed using Curves adjustment menu and Channel mixers adjustment menu in Photoshop CC 2017 (Adobe Systems) to improve contrast and filter out blue color background, respectively.

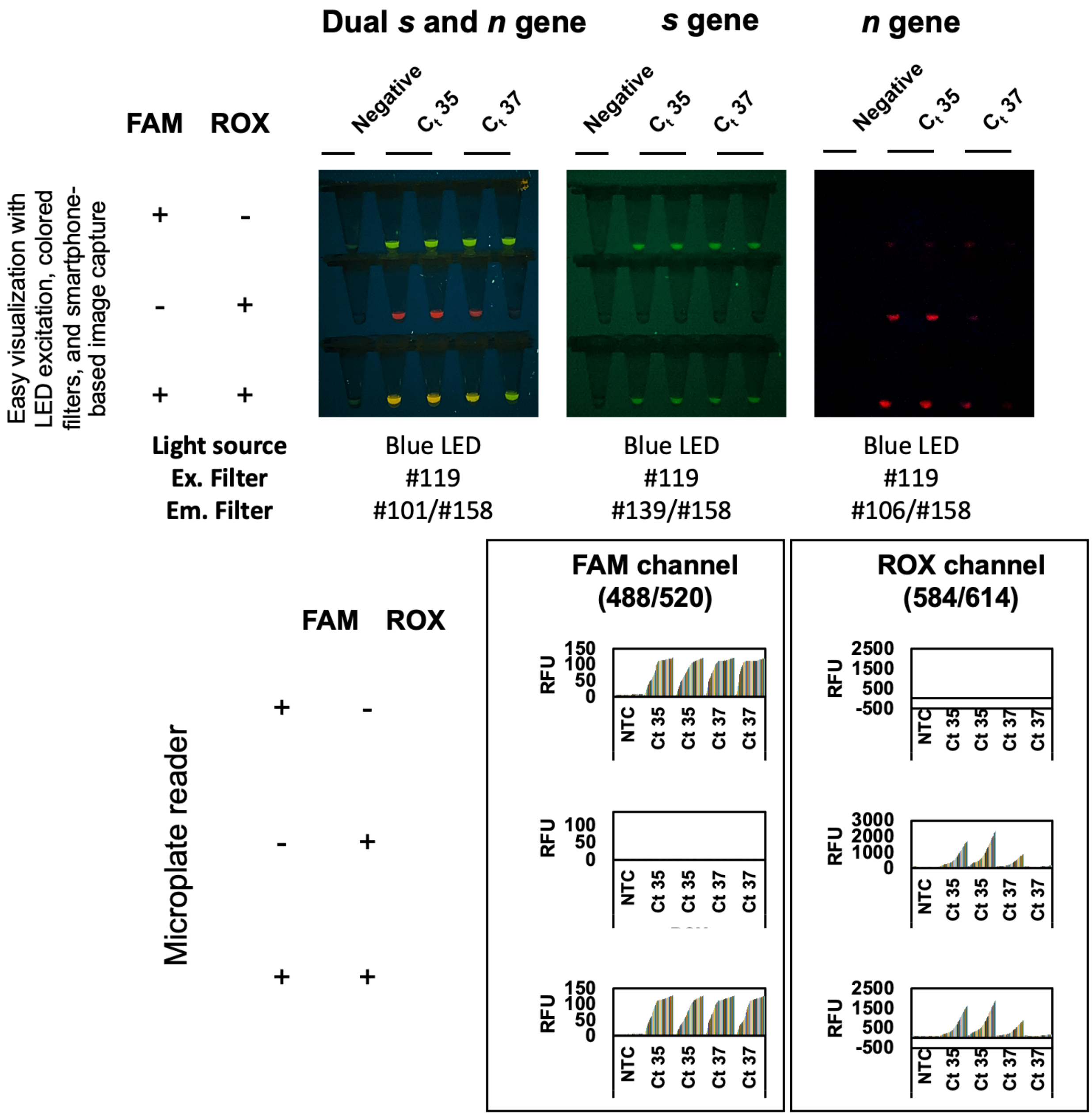

Figure S25. Matched sensitivity of the equipment-minimal approach of visualization with the microplate reader-based measurements. Multiplexed LwaCas13a-mediated *s*- and PsmCas13b-mediated *n*-gene detection was performed using diluted SARS-CoV-2 RNA (C_t 35 and 37, two replicates each) as input to the RT-RPA reactions. Reactions were monitored on a microplate reader over 120 min, then imaged using blue/white LED transilluminator excitation, lighting gels for emission filtration, and smartphone-based image capture. The ROX signal for one C_t 37 sample is very weak due to the amount of RNA input being near the limit of detection.

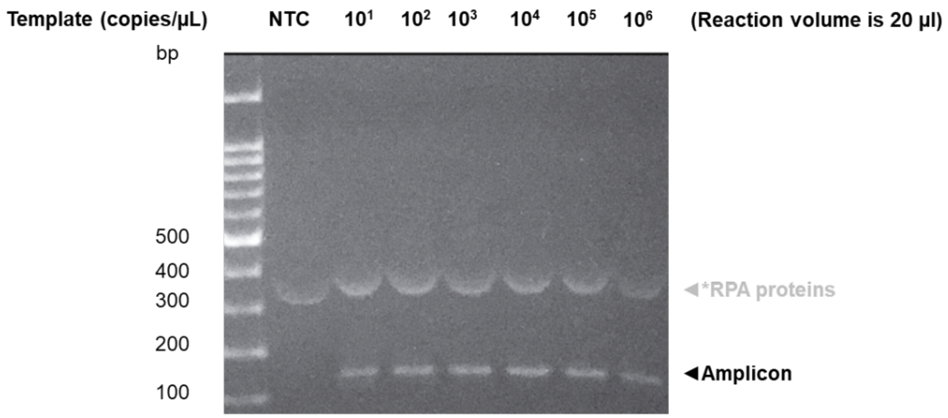

Figure S26: Activity of in-house RPA under standard reaction conditions.
RPA amplification reactions using in-house enzyme components were performed based on the standard reaction condition as described in the methods section. The copy number of the DNA template (T7 promoter linked to the SARS-CoV-2 *s* gene) used as input was varied as indicated. NTC is a negative control with RNase-free water as input. After incubation at 37°C for 60 min, RPA products were mixed with Novel Juice DNA staining reagent (BIO-HELIX), separated on 2% (w/v) agarose gel and visualized under blue light (BluPAD, BIO-HELIX). Positions of DNA template (T7 promoter-*s* gene, 137 bp) and amplicons were indicated with black arrowheads. The Novel Juice DNA stain also weakly stains proteins present in the reaction (white arrowheads).

A

	qPCR Ct					Copies per µL input				
**Dilution**	**Triplicates**			**Mean**	**SD**	**Triplicates**			**Mean**	**SD**
1x	27.15	27.20	27.23	27.19	0.04	912	896	984	930.67	46.88
10x	30.31	30.30	30.30	30.30	0.01	114.8	137.2	123.6	125.20	11.29
100x	34.12	33.75	33.86	33.91	0.19	8.4	12.4	12	10.93	2.20
1000x	38.30	38.25	37.36	37.97	0.53	1.12	0.4	2.4	1.31	1.01

B

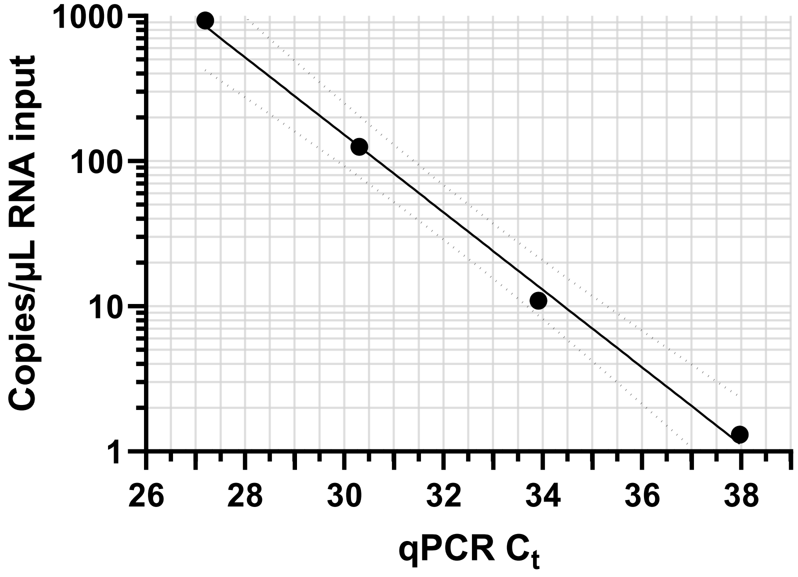

Figure S27: The linear correlation between qPCR Ct value and RNA copy number obtained from ddPCR. RT-qPCR or ddPCR analyses were performed on paired samples from a dilution series of RNA extract from the cultured SARS-CoV-2 Wuhan strain. The experiment was carried out in triplicate which the raw data are shown in **(A)** and the standard plot of qPCR Ct value versus copies per µL obtained from ddPCR is shown in **(B)**. Linear regression equation: $\log_{10}$ (Copies per µL input) = -0.2669 (qPCR C_t) + 10.187, $R^2 = 0.9967$. *Dotted lines represent the 95% confidence intervals.*

Table S1. Nomenclature of the SARS-CoV-2 lineages referenced in this study

This study	WHO label	NextStrain clade	PANGO lineage
Alpha	Alpha	20I (Alpha, V1)	B.1.1.7
Beta	Beta	20H (Beta, V2)	B.1.351
Gamma	Gamma	20J (Gamma, V3)	P.1
Delta	Delta	21A (Delta)<br>21I (Delta)<br>20J (Delta)	B.1617.2
Omicron	Omicron	21K (Omicron)<br><br>21L (Omicron)<br>20M (Omicron)	BA.1<br>BA.1.1<br>BA.2<br>BA.3

Table S2. Oligonucleotides used in this study

Name	Sequence	Sources	Ref.
**RT-RPA primer**			
SL_s-RPA-Forward_v1	gaaattaatacgactcactatagggAGGTTTCAAACTTTACTTGCTTTACATAGA	IDT	Patchsung et al., 2020
SL_s-RPA-Reverse_v1	TCCTAGGTTGAAGATAACCCACATAATAAG	IDT	Patchsung et al., 2020
SL_orf1a-RPA-Forward_v1	gaaattaatacgactcactatagggCGAAGTTGTAGGAGACATTATACTTAAACC	IDT	Patchsung et al., 2020
SL_orf1a-RPA-Reverse_v1	TAGTAAGACTAGAATTGTCTACATAAGCAGC	IDT	Patchsung et al., 2020
SI_orf1b-RPA-Forward_v1	gaaattaatacgactcactatagggTGATGCCATGCGAAATGCTGGTATTGTTGG	IDT	Patchsung et al., 2020
SI_orf1b-RPA-Reverse_v1	CTGCAGTTAAAGCCCTGGTCAAGGTTAATA	IDT	Patchsung et al., 2020
SI_n-RPA-Forward_v1	gaaattaatacgactcactatagggCGGCAGTCAAGCCTCTTCTCGTTCCTCATC	IDT	Patchsung et al., 2020
SI_n-RPA-Reverse_v1	CAGACATTTTGCTCTCAAGCTGGTTCAATC	IDT	Patchsung et al., 2020
SI-n-RPA-Forward_v2	gaaattaatacgactcactatagggTTCTCGTTCCTCATCACGTAGTCGCAACAG	IDT	This study
SI-n-RPA-Forward_v3	gaaattaatacgactcactatagggAACAGTTCAAGAAATTCAACTCCAGGCAGC	IDT	This study
SI-n-RPA-Forward_v4	gaaattaatacgactcactatagggGAACTTCTCCTGCTAGAATGGCTG	IDT	This study
SI-n-RPA-Forward_v5	gaaattaatacgactcactatagggGAACTTCTCCTGCTAGAATGGCTGGCAATG	IDT	This study
SI-n-RPA-Forward_v6	gaaattaatacgactcactatagggCTAGAATGGCTGGCAATGGCGGTGATGCTG	IDT	This study
SGF3675-3677Del_F	gaaattaatacgactcactataggAGTTGGGTGATGCGTATTATGACATGGTTG	IDT	This study
SGF3675-3677Del_R	AGGATTAGTAACACTACAGCTGATGCATAC	IDT	This study
HV69-70Del_F	gaaattaatacgactcactataggATTACCCTGACAAAGTTTTCAGATCCTCAG	IDT	This study
HV69-70Del_R	ATAAACACCATCATTAAATGGTAGGACAGG	IDT	This study
Y144Del_F	gaaattaatacgactcactataggACGCTACTAATGTTGTTATTAAAGTCTGTG	IDT	This study
Y144Del_R	AAAGTGCAATTATTCGCACTAGAATAAACT	IDT	This study
EF156-157Del_R	GGCTGAGAGACATATTCAAAAGTGCAATTA	IDT	This study
L452R_F	gaaattaatacgactcactataggACAGGCTGCGTTATAGCTTGGAATTCTAAC	IDT	This study
L452R_R	TGAGATTAGACTTCCTAAACAATCTATACC	IDT	This study
**crRNA**			
13a_SL_s-crRNA_v1	gauuuagacuaccccaaaaacgaaggggacuaaaacGCAGCACCAGCUGUCCAACCUGAAGAAG	Synthego	Patchsung et al., 2020
13a_SL_orf1a-crRNA_v1	gauuuagacuaccccaaaaacgaaggggacuaaaacCCAACCUCUUCUGUAAUUUUAAACUAU	Synthego	Patchsung et al., 2020

**Name**	**Sequence**	**Sources**	**Ref.**
13a_SI_orf1b-crRNA_v1	gauuuagacuaccccaaaaacgaaggggacuaaaacGGAACUCCACUACCUGGCGUGGUUUGUA	Synthego	Patchsung et al., 2020
13a_SI_n-crRNA_v1	gauuuagacuaccccaaaaacgaaggggacuaaaacAAAGCAAGAGCAGCAUCACCGCCAUUGC	Synthego	Patchsung et al., 2020
Psm13b_SI_n-crRNA-v1	aaagcaagagcagcaucaccgccauugccaGUUGUAGAAGCUUAUCGUUUGGAUAGGUAUGACAAC	Genscript	This study
**crRNA template for IVT**			
SGF3675-3677Del	ATACTAGTTTGAAGCTAAAAGACTGTGTgttttagtccccttcgtttttggggtagtctaaatcCCTATAGTGAGTCGTATTAATTTC	IDT	This study
HV69-70Del	TTGGTTCCATGCTATCTCTGGGACCAATgttttagtccccttcgttttggggtagtctaaatcCCTATAGTGAGTCGTATTAATTTC	IDT	This study
Y144Del	TTTTGGGTGTTTACCACAAAAACAACAAgttttagtccccttcgtttttggggtagtctaaatcCCTATAGTGAGTCGTATTAATTTC	IDT	This study
EF156-157Del	AAGTTGGATGGAAAGTGGAGTTTATTCTgttttagtccccttcgtttttggggtagtctaaatcCCTATAGTGAGTCGTATTAATTTC	IDT	This study
L452R	AAGGTTGGTGGTAATTATAATTATCGGTgttttagtccccttcgtttttggggtagtctaaatcCCTATAGTGAGTCGTATTAATTTC	IDT	This study
**Reporter**		IDT	
Cas13a_reporter	/FAM/mArArUrGrGrCmAmArArUrGrGrCmA/Bio/	IDT	Patchsung et al., 2020
FAM PolyU reporter	/FAM/rUrUrUrUrUrC/IABkFQ/	IDT	Patchsung et al., 2020
Cy5 PolyA reporter	/Cy5/rArArArArA/IAbRQSp/	IDT	This study
ROX PolyA reporter	/ROX/rArArArArA/IAbRQSp/	IDT	This study

Table S3. Specific RT-RPA conditions for each figure. Aside from parameters shown in the table which were varied during optimizations, all other parameters are as given in the "Optimized multiplexed RT-RPA" methods section.

Figure #	Primer identity and concentrations	RNase H Concentrations	Additive	RNA input
Figure 2A	177 nM each *s* primer and 222 nM *n* primer	0.025 U/μL	40 mM Triglycine	5.3
Figure 2B-2E	177 nM each *s* primer and 222 nM n primer	0.025 U/μL	40 mM Triglycine	12.4
Figure 2F	177 nM each *s* primer and 222 nM *n* primer	0.025 U/μL	40 mM Triglycine	12.4
Figure 3A-3B	704 nM each primer for each variant	0.025 U/μL	40 mM Triglycine	12.4
Figure 3C	177 nM each *s*(EF156/157ΔR158G) primer and 177 nM *n* primer	0.025 U/μL	40 mM Triglycine	12.4
Figure 3D	177 nM each *s*(EF156/157ΔR158G) primer and 177 nM *n* primer	0.025 U/μL	40 mM Triglycine	12.4
Figure 3E	177 nM each HV69/70Δ primer and 177 nM *n* primer	0.025 U/μL	40 mM Triglycine	12.4
Figure 3F	177 nM each HV69/70Δ primer and 177 nM *n* primer	0.025 U/μL	40 mM Triglycine	12.4
Figure 4A	177 nM each *s* primer and 222 nM *n* primer	0.025 U/μL	40 mM Triglycine	5.3
Figure S1C	709 nM each *n* primer	-	-	6.3 μL
Figure S1D	355 nM each primer for each gene	-	-	6.3 μL
Figure S2A, 2B	varying concentration of *s* and N primer pair	0.025 U/μL	-	6.3 μL
Figure S2C	709 nM each *s* primer	Varying RNase H concentrations	-	6.3 μL

Figure #	Primer identity and concentrations	RNase H Concentrations	Additive	RNA input
Figure S2D	704 nM each *n* primer, 704 each *s* primer, and 177 nM each *s* priemr and 222 nM *n* primer for s, n and s+n respectively	0.025 U/µL	40 mM Triglycine	5.3 µL
Figure S2E	704 nM each *n* primer	0.025 U/µL	40 mM Triglycine	5.3 µL
Figure S4	709 nM each *n* primer	-	-	6.3 µL
Figure S5	709 nM each *n* primer	1 U/µL	-	6.3 µL
Figure S6	709 nM each *n* primer	-	-	6.3 µL
Figure S7A	177 nM each *s* and *n* primer	0.025 U/µL	Varying additives	3.2 µL
Figure S7B	177 nM each *s* and *n* primer	0.025 U/µL	-	6.3 µL
Figure S7C	177 nM each *s* and *n* primer	0.025 U/µL	With and without 40 mM Triglycine	3.2 µL
Figure S8	177 nM each *s* primer and 222 nM *n* primer	0.025 U/µL	40 mM Triglycine	5.3 µL
Figure S10	704 nM each *n* primer	0.025 U/µL	40 mM Triglycine	5.3 µL
Figure S11	177 nM each *s* primer and 222 nM *n* primer	0.025 U/µL	40 mM Triglycine	5.3 µL
Figure S12	177 nM each s primer and 222 nM *n* primer	0.025 U/µL	40 mM Triglycine	12.4 µL
Figure S16	704 nM each primer for each variant	0.025 U/µL	40 mM Triglycine	12.4 µL
Figure S17	704 nM each *s*(EF156/157ΔR158G) primer	0.025 U/µL	40 mM Triglycine	12.4 µL
Figure S18	177 nM each *s*(EF156/157ΔR158G or HV69/70Δ) primer and 177 nM *n* primer	0.025 U/µL	40 mM Triglycine	12.4 µL
Figure S19	704 nM each *s*(HV69/70Δ) primer	0.025 U/µL	40 mM Triglycine	12.4 µL

Table S4.Specific Cas13-based reaction conditions for each figure. Aside from parameters shown in the table which were varied during optimizations, all other parameters are as given in the "Optimized multiplexed Cas13-based detection with fluorescence readout" methods section.

**Figure**	**Buffer identity and concentration**	**Cas enzyme identity and concentration**	**crRNA identity and concentration**	**RNA reporter identity and concentration**	**additive**	**$MgCl_2$ concentration**	**Equipment used to monitor Cas-based reactions**
Figure 2 Multiplexed LwaCas13a-PsmCas13b detection	20 mM HEPES pH 6.8	45 nM LwaCas13a, 135 nM PsmCas13b,	22.5 nM LwaCas13a crRNA targeting *s* gene, 67.5 nM PsmCas13b crRNA targeting *n* gene	250 nM PolyU-FAM, 500 nM PolyA-ROX	750 mM betaine	24 mM	Tecan microplate reader
Figure 2 LwaCas13a Dual *s*/*n* detection	20 mM HEPES pH 6.8	45 nM LwaCas13a	22.5 nM LwaCas13a crRNA targeting *s* gene, 22.5 nM LwaCas13a crRNA targeting *n* gene	250 nM PolyU-FAM	750 mM betaine	24 mM	Tecan microplate reader
Figure 3A-3B	40 mM Tris pH 7.4	45 nM LwaCas13a	22.5 nM LwaCas13a crRNA targeting each variant	250 nM PolyU-FAM	-	6 mM	Tecan microplate reader
Figure 3C	40 mM Tris pH 7.4	45 nM LwaCas13a, 135 nM PsmCas13b,	22.5 nM LwaCas13a crRNA targeting each variant, 67.5 nM PsmCas13b crRNA targeting *n* gene	250 nM PolyU-FAM, 500 nM PolyA-ROX	750 mM betaine	24 mM	Tecan microplate reader
Figure 3D	40 mM Tris pH 7.4	45 nM LwaCas13a,	22.5 nM LwaCas13a crRNA targeting	250 nM PolyU-FAM,	750 mM betaine	24 mM	Tecan microplate reader

Figure	Buffer identity and concentration	Cas enzyme identity and concentration	crRNA identity and concentration	RNA reporter identity and concentration	additive	$MgCl_2$ concentration	Equipment used to monitor Cas-based reactions
		135 nM PsmCas13b	each variant, 67.5 nM PsmCas13b crRNA targeting *n* gene	500 nM PolyA-ROX			
Figure 3E	40 mM Tris pH 6.8	45 nM LwaCas13a, 135 nM PsmCas13b	22.5 nM LwaCas13a crRNA targeting HV69/70Δ, 67.5 nM PsmCas13b crRNA targeting *n* gene	250 nM PolyU-FAM, 500 nM PolyA-ROX	-	24 mM	Tecan microplate reader
Figure 3F	40 mM Tris pH 6.8	45 nM LwaCas13a, 135 nM PsmCas13b	22.5 nM LwaCas13a crRNA targeting HV69/70Δ, 67.5 nM PsmCas13b crRNA targeting *n* gene	250 nM PolyU-FAM, 500 nM PolyA-ROX	-	24 mM	Tecan microplate reader
Figure 4A	40 mM Tris pH 7.4	45 nM LwaCas13a	22.5 nM LwaCas13a crRNA targeting *s* gene, 22.5 nM LwaCas13a crRNA targeting *n* gene	250 nM PolyU-FAM	-	6 mM	Real-time PCR
Figure 4B	20 mM HEPES pH 6.8	45 nM LwaCas13a, 135 nM PsmCas13b,	22.5 nM LwaCas13a crRNA targeting *s* gene, 67.5 nM PsmCas13b crRNA targeting *n* gene	Only 250 nM PolyU-FAM, only 500 nM PolyA-ROX, or both	750 mM betaine	24 mM	Naked eye visualization

**Figure**	**Buffer identity and concentration**	**Cas enzyme identity and concentration**	**crRNA identity and concentration**	**RNA reporter identity and concentration**	**additive**	**$MgCl_2$ concentration**	**Equipment used to monitor Cas-based reactions**
Figure S1C, 1D	40 mM Tris pH 7.4	45 nM LwaCas13a	22.5 nM LwaCas13a crRNA for each gene	1 µM FAM-Bio	-	6 mM	Lateral flow strip
Figure S2A, 2B, 2D	20 mM HEPES pH 6.8	45 nM LwaCas13a, 135 nM PsmCas13b,	22.5 nM LwaCas13a crRNA targeting *s* gene, 67.5 nM PsmCas13b crRNA targeting *n* gene	250 nM PolyU-FAM, 500 nM PolyA-Cy5	-	24 mM	Tecan microplate reader
Figure 2C	40 mM Tris pH 7.4	45 nM LwaCas13a	22.5 nM LwaCas13a crRNA for *s* gene	250 nM PolyU-FAM	-	6 mM	Tacan microplate reader
Figure 4B	20 mM HEPES pH 6.8	45 nM LwaCas13a, 135 nM PsmCas13b,	22.5 nM LwaCas13a crRNA targeting *s* gene, 67.5 nM PsmCas13b crRNA targeting *n* gene	Only 250 nM PolyU-FAM, only 500 nM PolyA-ROX, or both	750 mM betaine	24 mM	Naked eye visualization
Figure 4D, 4E, and 4F	40 mM Tris pH 7.4	45 nM LwaCas13a	22.5 nM LwaCas13a crRNA for *n* gene	250 nM PolyU-FAM	-	6 mM	Tacan microplate reader
Figure 4G	40 mM Tris pH 7.4	45 nM LwaCas13a	22.5 nM LwaCas13a crRNA targeting *s* gene, 22.5 nM LwaCas13a crRNA targeting *n* gene	250 nM PolyU-FAM	-	6 mM	Real-time PCR
Figure 4H	40 mM Tris pH 7.4	45 nM LwaCas13a	22.5 nM LwaCas13a	250 nM PolyU-FAM	-	6 mM	Real-time PCR

Figure	Buffer identity and concentration	Cas enzyme identity and concentration	crRNA identity and concentration	RNA reporter identity and concentration	additive	$MgCl_2$ concentration	Equipment used to monitor Cas-based reactions
			crRNA targeting *s* gene, 22.5 nM LwaCas13a crRNA targeting *n* gene				
Figure S1	40 mM Tris pH 7.4	45 nM LwaCas13a	22.5 nM LwaCas13a crRNA for each gene	1 µM FAM-Bio	-	6 mM	Lateral flow strip
Figure S1D	40 mM Tris pH 7.4	45 nM LwaCas13a	22.5 nM LwaCas13a crRNA for each gene	1 µM FAM-Bio	-	6 mM	Lateral flow strip
Figure S2A, 2B, 2D	20 mM HEPES pH 6.8	45 nM LwaCas13a, 135 nM PsmCas13b,	22.5 nM LwaCas13a crRNA targeting *s* gene, 67.5 nM PsmCas13b crRNA targeting *n* gene	250 nM PolyU-FAM, 500 nM PolyA-Cy5	-	24 mM	Tecan microplate reader
Figure S2C	40 mM Tris pH 7.4	45 nM LwaCas13a	22.5 nM LwaCas13a crRNA for *s* gene	250 nM PolyU-FAM	-	6 mM	Tacan microplate reader
Figure S2D	40 mM Tris pH 7.4	45 nM LwaCas13a	22.5 nM LwaCas13a crRNA for *s* gene or *n* gene	250 nM PolyU-FAM	-	6 mM	Tacan microplate reader
Figure S2E	40 mM Tris pH 7.4	45 nM LwaCas13a	22.5 nM LwaCas13a crRNA for *n* gene	250 nM PolyU-FAM	-	6 mM	Tacan microplate reader
Figure S4A	20 mM HEPES pH 6.8	Varying PsmCas13b amount	Varying PsmCas13b crRNA	250 nM PolyA-Cy5	-	6 mM	Varioskan microplate reader

Figure	Buffer identity and concentration	Cas enzyme identity and concentration	crRNA identity and concentration	RNA reporter identity and concentration	additive	$MgCl_2$ concentration	Equipment used to monitor Cas-based reactions
Figure S4B	20 mM HEPES pH 6.8	135 nM PsmCas13b	67.5 nM PsmCas13b crRNA	varying PolyA-Cy5 amount	-	6 mM	Varioskan microplate reader
Figure S5 LwaCas13a	20 mM HEPES pH 6.8	45 nM LwaCas13a	22.5 nM LwaCas13a crRNA for *n* gene	250 nM PolyU-FAM	-	6 mM	Varioskan microplate reader
Figure S5 PsmCas13b	20 mM HEPES pH 6.8	135 nM PsmCas13b	67.5 nM PsmCas13b crRNA	500 nM PolyA-Cy5	-	6 mM	Varioskan microplate reader
Figure S6B	40 mM Tris pH 7.4	45 nM RfxCas13d	28.4 nM RfxCas13d crRNA	250 nM PolyU-FAM reporter or 125 nM RNase Alert	-	6 mM	Varioskan microplate reader
Figure S6C	40 mM Tris pH 7.4	45 nM RfxCas13d or RfxCas13d-RBD or LwaCas13a	22.5 nM LwaCas13a crRNA or 28.4 nM RfxCas13d crRNA	500 nM PolyU/FAM reporter	-	6 mM	Tecan microplate reader
Figure S7A	40 mM Tris pH 7.4	45 nM LwaCas13a	22.5 nM LwaCas13a crRNA for target gene	250 µM PolyU-FAM	-	6 mM	Tacan microplate reader
Figure S7B	20 mM HEPES pH 6.8	45 nM LwaCas13a, 135 nM PsmCas13b,	22.5 nM LwaCas13a crRNA targeting *s* gene, 67.5 nM PsmCas13b crRNA targeting *n* gene	250 nM PolyU-FAM, 500 nM PolyA-Cy5	Varying additives	24 mM	Tecan microplate reader
Figure S7C	20 mM HEPES pH 6.8	45 nM LwaCas13a,	22.5 nM LwaCas13a crRNA targeting *s*	250 nM PolyU-FAM,	With and without	24 mM	Tecan microplate reader

Figure	Buffer identity and concentration	Cas enzyme identity and concentration	crRNA identity and concentration	RNA reporter identity and concentration	additive	$MgCl_2$ concentration	Equipment used to monitor Cas-based reactions
		135 nM PsmCas13b,	gene, 67.5 nM PsmCas13b crRNA targeting *n* gene	500 nM PolyA-Cy5	500 mM betaine		
Figure S8	20 mM HEPES pH 6.8	45 nM LwaCas13a, 135 nM PsmCas13b,	22.5 nM LwaCas13a crRNA targeting *s* gene, 67.5 nM PsmCas13b crRNA targeting *n* gene	250 nM PolyU-FAM, 500 nM PolyA-Cy5	Varying betaine amount	24 mM	Tecan microplate reader
Figure S9A	40 mM Tris pH 7.4	Varying amount of LwaCas13a	Varying amount of LwaCas13a crRNA targeting *s* gene	250 nM PolyU-FAM	-	6 mM	RT-PCR machine
Figure S9B	20 mM HEPES pH 6.8	Varying amount of PsmCas13b	Varying amount of PsmCas13b crRNA targeting *n* gene	500 nM PolyA-Cy5	-	6 mM	Varioskan microplate reader
Figure S9C	Varying type of buffers	45 nM LwaCas13a, 45 nM PsmCas13b	45 nM LwaCas13a crRNA targeting *s* gene, 45 nM PsmCas13b crRNA targeting *n* gene	250 nM PolyU-FAM, 500 nM PolyA-Cy5	-	6 mM	Tecan microplate reader
Figure S10	40 mM Tris pH 7.4	45 nM LwaCas13a	22.5 nM LwaCas13a crRNA targeting *n* gene		-	6 mM	Real-time PCR
Figure S11	40 mM Tris pH 7.4	45 nM LwaCas13a	22.5 nM LwaCas13a crRNA targeting *s*	250 nM PolyU-FAM	750 mM betaine	24 mM	Tecan microplate reader

**Figure**	**Buffer identity and concentration**	**Cas enzyme identity and concentration**	**crRNA identity and concentration**	**RNA reporter identity and concentration**	**additive**	**$MgCl_2$ concentration**	**Equipment used to monitor Cas-based reactions**
			gene, 22.5 nM LwaCas13a crRNA targeting *n* gene (s/n gene detection)				
Figure S22	20 mM HEPES pH 6.8	45 nM LwaCas13a, 135 nM PsmCas13b,	22.5 nM LwaCas13a crRNA targeting *s* gene, 67.5 nM PsmCas13b crRNA targeting *n* gene	250 nM PolyU-FAM with 500 nM PolyA-Cy5, 250 nM or 500 nM PolyA-ROX	750 mM betaine	24 mM	Tecan microplate reader

Table S5. The limit of detection (LoD) of the multiplexed CRISPR-based detection of SARS-CoV-2 RNA. Estimated LoDs at 95% positive rate (95% LoD) from LwaCas13a-mediated *s* gene detection, PsmCas13b-mediated *n* gene detection, LwaCas13a-mediated *s* and *n* gene detection, or combined LwaCas13a-mediated *s* gene and PsmCas13b-mediated *n* gene detection determined using Probit regression. Calculation was performed using MedCalc (MedCalc software Ltd).

**95% LoD qPCR C_t (95% CI)**	**At 60 minutes CRISPR reaction time**
**Detecting the *s* gene via LwaCas13a**	36.83*
**Detecting the *n* gene via PsmCas13b**	36.78*
**Detecting either the *s* or *n* gene via LwaCas13a**	36.48 (35.99 – 36.97)
**Detecting either the *s* or *n* gene via LwaCas13a/PsmCas13b**	37.01*

*The margins of error were negligible.
